# Wearable Electronic Monitoring of Vital Signs in Hospitalised Adults: A Nursing Focused Scoping Review of Clinical, Economic and Implementation Outcomes

**DOI:** 10.1111/jan.70583

**Published:** 2026-03-26

**Authors:** Sian Myfanwy Shaw, Sophie Bethan Shaw, Gillian Janes

**Affiliations:** ^1^ School of Nursing Anglia Ruskin University Cambridge UK; ^2^ Departments of Psychology and Economics University of Bath Bath UK

**Keywords:** continuous vital signs monitoring, nursing workflow, patient experience, patient safety, scoping review, wearable technology

## Abstract

**Aim:**

To synthesise evidence on wearable devices for continuous vital signs monitoring in adult hospital inpatients, focusing on clinical effectiveness, nursing perspectives, workflow impact, patient experience and resource implications.

**Design:**

Scoping review.

**Review Methods:**

Joanna Briggs Institute methodology reported using PRISMA‐ScR guidelines.

**Data Sources:**

Six databases (CINAHL, MEDLINE, EMBASE, Scopus, Web of Science, Cochrane), Scite.ai, and hand searching for studies published between January 2015 and November 2025. Data were synthesised using reflexive thematic analysis.

**Results:**

Sixty‐seven studies from 19 countries were included. Four integrative themes were identified. (1) Enhancing clinical safety through continuous monitoring: wearable devices consistently enable earlier recognition of physiological deterioration; however, downstream outcomes such as length of stay and transfers to intensive care units were mixed and context dependent. (2) Transforming nursing practice and workflow integration highlighted improved situational awareness and potential efficiency gains, alongside challenges related to alarm overload, parallel documentation and implementation workload. (3) *Patient experience of wearable monitoring*: most patients reported reassurance and perceived safety, though experiences reflected a tension between monitoring as care and monitoring as surveillance; discomfort, anxiety, and privacy considerations were infrequently examined. (4) *Economic and organisational consequences*: potential system value was suggested through workforce efficiencies, but economic benefits were largely inferred, with infrastructure and training costs often underreported.

**Conclusion:**

Wearable continuous monitoring technologies show clear potential to support nursing observations enabling improved early detection of deterioration. Realising these benefits depends on effective integration into workflows, robust governance, and sustained nursing leadership rather than technological capability alone. Significant evidence gaps remain regarding long‐term outcomes, economic evaluation, and large‐scale implementation.

**Impact:**

Wearable devices for continuous vital signs monitoring have the potential to transform inpatient surveillance by enabling earlier recognition of physiological deterioration and enhancing nurses' situational awareness. This scoping review synthesises international evidence demonstrating that, although wearable monitoring can improve patient safety and workflow efficiency, its impact depends on effective integration into nursing practice, governance structures, and organisational preparedness. Continuous monitoring also introduces new challenges including alert fatigue, data interpretation, and workflow redesigns, highlighting the vital role of nursing leadership in digital health implementation. The review also identifies critical evidence gaps, particularly concerning long‐term clinical outcomes, patient experience, and cost‐effectiveness, providing priorities for future research and policy to promote safe, ethical, and sustainable adoption.

**Patient or Public Involvement:**

None.

**What does this paper contribute to the wider global clinical community?**

- Synthesises international evidence showing that wearable continuous vital signs monitoring improves early recognition of patient deterioration, while explaining why downstream outcomes such as intensive care transfer and length of stay remain inconsistent across settings.
- Demonstrates that the impact of wearable monitoring depends on how technologies are embedded within nursing workflows, positioning nursing practice and leadership as central to safety, effectiveness, and sustainability.
- Advances understanding of alert fatigue by framing it not only as an operational burden but as a challenge to professional judgement, trust in technology and clinical sense‐making.
- Integrates patient experience into the evidence base, highlighting reassurance and acceptance alongside underexplored concerns related to discomfort, anxiety, trust, and surveillance.
- Clarifies that economic value is largely inferred rather than demonstrated, identifies critical gaps in cost‐effectiveness evidence, and underscores the importance of workforce sustainability and system readiness in digital health adoption.

## 1 Introduction

Escalating economic pressures, patient acuity and global nursing shortages are straining healthcare systems, challenging the viability of traditional inpatient care models (Darzi 2024; World Health Organization 2024). These pressures have heightened demands for patient safety, workforce resilience and efficiency, driving interest in technology‐enabled, person‐centred care. International and national policies, including the World Health Organization (2024) Global Initiative on Digital Health, the United Nations (2022) Digital Strategy, and the National Health Service (NHS) Long‐Term Plan (UK Government 2025), highlight digital transformation as essential to delivering safe, high‐quality and sustainable healthcare. Within these policies, digital monitoring and data‐driven decision‐making are identified as key to early detection of patient deterioration and to optimising resource utilisation.

Wearable devices for continuous vital signs monitoring are a key innovation. These compact, wireless, medical‐grade technologies enable ongoing measurement of parameters such as heart rate (HR), respiratory rate (RR), peripheral oxygen saturation (SpO_2_), blood pressure (BP) and temperature, with minimal disruption to patients (Dias and Cunha 2018). In community‐based care and virtual wards, they assist in managing chronic diseases, enable early intervention and help reduce hospital readmissions (Kirk et al. 2018; NHS England 2023). Despite these advantages, their use in acute hospital inpatient settings remains limited.

In hospital care, monitoring vital signs is a fundamental aspect of patient surveillance and clinical decision‐making, mainly performed intermittently by nurses and healthcare assistants, usually every 4–6 h on general wards and more frequently for patients who are postoperative or at risk of clinical deterioration (Considine et al. 2023; Redfern et al. 2019). While intermittent monitoring supports routine assessment, it may miss rapid or brief physiological changes, especially in urgent conditions such as sepsis or respiratory failure (Mok et al. 2015). Evidence indicates that changes in vital signs are strong indicators of adverse outcomes, including admission to the intensive care unit (ICU) and in‐hospital mortality (Barfod et al. 2012; Brekke et al. 2019; Choi et al. 2022). However, early warning systems based on intermittent measurements often fail to detect severe abnormalities, thereby reducing their ability to prevent deterioration (Haahr‐Raunkjaer, Mølgaard, et al. 2022). Nurses and midwives are ideally placed to help improve health outcomes through digital healthcare transformation, but their policy leadership potential is underused (Janes et al. 2024).

Continuous vital signs monitoring has the potential to overcome these limitations by allowing real‐time data collection and trend analysis. Studies indicate that continuous monitoring systems can detect physiological abnormalities several hours earlier than intermittent methods, depending on the technology and clinical setting (Churpek et al. 2016). Transient but clinically important changes, such as episodic hypoxaemia, may occur between scheduled observations and go unnoticed under intermittent monitoring regimens, thereby delaying necessary care escalation (Leenen et al. 2020; Saab et al. 2021). Since deterioration often follows detectable physiological patterns, sometimes hours before critical events like cardiac arrest, there is a growing recognition of the importance of monitoring systems capable of continuous assessment (Leenen, Dijkman, et al. 2022).

For nursing, the implications of continuous monitoring extend beyond technological capabilities to encompass professional practice, workflow and organisational systems. Nurses constitute the largest segment of the global healthcare workforce (World Health Organization 2020) and play a vital role in patient surveillance, interpretation of physiological data, and care escalation. Manual vital signs monitoring remains a fundamental nursing responsibility but is highly resource‐intensive, with each set of observations estimated to take approximately 5 min to complete and record (Dall'Ora et al. 2021). When applied across inpatient populations, this results in a significant cumulative workload and cost burden, exacerbated by workforce shortages expected to exceed 4.5 million nurses worldwide by 2030 (World Health Organization 2024).

Although wearable continuous monitoring systems may reduce the workload of routine observations and enhance situational awareness, they also introduce new challenges related to workflow integration, alarm management, relational care, professional judgement and clinical governance. Understanding how these technologies influence nursing practice and leadership is therefore vital to ensuring safe, effective and person‐centred implementation. To date, evidence syntheses examining continuous vital signs monitoring have largely focused on technical performance, feasibility, or specific patient populations, with limited consideration of nursing, organisational and experiential outcomes. Reviews by Downey, Chapman, et al. (2018), Leenen et al. (2020), and Huhn et al. (2022) offer valuable insights into the potential of wireless and digital monitoring technologies but do not comprehensively map their multidimensional impacts across patient safety, nursing practice, patient experience, and resource utilisation. As wearable monitoring technologies move from pilot studies to routine clinical adoption, a broader synthesis of the evidence is needed to inform nursing leadership, service design and policy.

Accordingly, this scoping review aims to map and synthesise the international evidence on the use of wearable devices for continuous vital signs monitoring in adult hospital inpatients. In line with PRISMA‐ScR guidance, the review seeks to examine the extent, range and nature of the evidence to strengthen the evidence base underpinning safe, person‐centred and sustainable digital transformation in acute hospital settings and to identify priorities for future research, leadership and policy development.

## 2 The Review

### 2.1 Aim and Objectives

This scoping review synthesises the existing evidence on wearable devices for continuous vital signs monitoring in adult hospital inpatients, focusing on:

1. Clinical effectiveness and patient safety.
2. Nursing perspectives and workflow impact.
3. Patient acceptance and experience.
4. Resource Implications.

### 2.2 Method

#### 2.2.1 Design

A scoping review, based on Arksey and O'Malley's (2005) framework, which was updated by Munn et al. (2018), was chosen to guide synthesis of existing evidence, identification of gaps and concept clarification without evaluating evidence quality. This approach is exploratory, broad and flexible, making it suitable for the evolving field of wearable technology. This approach offers insights for clinicians, policymakers and researchers on improving patient outcomes, clinical workflow, economic factors and adoption barriers.

This study is reported in accordance with the PRISMA‐ScR guidelines (Tricco et al. 2016) and the Joanna Briggs Institute (JBI) scoping review guidelines (Peters et al. 2020).

#### 2.2.2 Search Methods

Two authors, advised by an academic librarian, developed a search strategy using the Population, Concept, and Context (PCC) framework (Richardson et al. 1995). The approach was refined through team discussions, combining MeSH Headings, keywords, and filters for each database (Table 1). After testing in one database, the final search was applied across six databases: CINAHL, MEDLINE (Ovid), EMBASE, Scopus, Web of Science and the Cochrane Database, covering publications from January 2015 to December 2024. The initial search was conducted in December 2024 and an update was conducted in November 2025 using the same criteria. This period marks the shift of wearable vital sign devices from prototypes to validated tools, with key developments such as consumer wearables with medical sensors (Apple Watch) (Romero‐Chan 2024), advances in Bluetooth for continuous monitoring (Koulouras et al. 2025), and FDA clearances for ECG and SpO_2_ devices (FDA 2025). The results were exported to RefWorks.

**TABLE 1:** Search strategy used for MEDLINE (Ovid).

Search ID no		Results
	*Population*	
1	“Inpatients” OR “Hospital? * Patients” OR “Critical Care Patient*” OR “Intensive Care Patient*” OR “Emergency Service, Hospital” OR “Surgical Patient*” OR “High Dependency Patient*” OR “Ward Patient*” OR “Medical” OR “Surgical”	9,932,127
	*Concept*	
2	“Wearable Device*” OR “Wearable Technology” OR “Wearable System*” OR “Monitoring, Physiologic” OR “Remote Monitoring” OR “Wearable Sensor*” OR “Mobile Health Device*” OR “Continuous Vital Sign Monitoring” OR “Wearable Patch” OR “Smart Watch?*“ OR “Apple Watch?*” OR “Mobile Monitoring” OR “Continuous Vital Sign Monitoring” OR “Wireless Monitoring” OR “Wearable Electronic Device” OR “Remote Monitoring”	79,691
	*Context*	
3	“Vital Sign*” OR “Observation*” OR “Blood Pressure” OR “Pulse” OR “Heart Rate” OR “emperature” OR “Oxygen Saturation” OR “SpO_2_” OR “Patient Monitoring”	2,995,512
	*Outcome*	
4	“Clinical Outcome*” OR “Mortality” OR “Morbidity” OR “Cost‐Benefit Analysis” OR “Economics, Medical” OR “Cost Saving*” OR “Patient Satisfaction” OR “Quality of Health Care” OR “Patient‐Reported Outcome Measure*” OR “Job Satisfaction” OR “Treatment Outcome*” OR “Complication* Postoperative” OR “Workload” OR “Workflow” OR “Nurs? Care” OR “Deteriorat?” OR “Readmission ” OR “ICU admission“	3,362,290
	*Combined search strategy*	
5	1 and 2 and 3 and 4	1618
	*Limits*	
6	Limit 5 to (English language and humans and yr = “2015 – 2025” and “all adult (19 plus years)“)	386

Specialist journals, such as *Nursing Informatics*, were also reviewed. This process was supplemented by citation tracking and screening of the reference lists of included studies. In addition, the smart citation index Scite.ai (Scite 2024) was integrated into the search strategy. SCITE.ai retrieves evidence‐based responses by referencing a large corpus of peer‐reviewed literature with direct links to full‐text sources (Lund and Shamsi 2023).

#### 2.2.3 Inclusion and Exclusion Criteria

The inclusion and exclusion criteria for selecting studies are outlined in Table 2.

**TABLE 2:** Inclusion and exclusion criteria.

Criteria	Inclusion	Exclusion
Population	Adult hospital inpatients in clinical settings such as ICU, postoperative wards, general inpatient care	Paediatric, veterinary populations, or outpatient/community care settings
Concept	Use of wearable devices for continuous monitoring of vital signs (HR, RR, SpO_2_, BP, Temperature)	Studies focusing on fitness trackers
Context	Clinical settings such as ICU, postoperative, general inpatient care, rehabilitation units	Non‐clinical settings or studies focusing on home‐based or remote monitoring without hospital context
Study design	Qualitative and quantitative research, including observational studies, clinical trials, pilot trials	Commentaries, editorials, opinion pieces, and studies without peer review, grey literature
Publication year	Studies published between Jan 2015 and Dec 2025	Studies published before 2015
Language	Studies published in English	Studies published in languages other than English

#### 2.2.4 Study Selection

**FIGURE 1:**
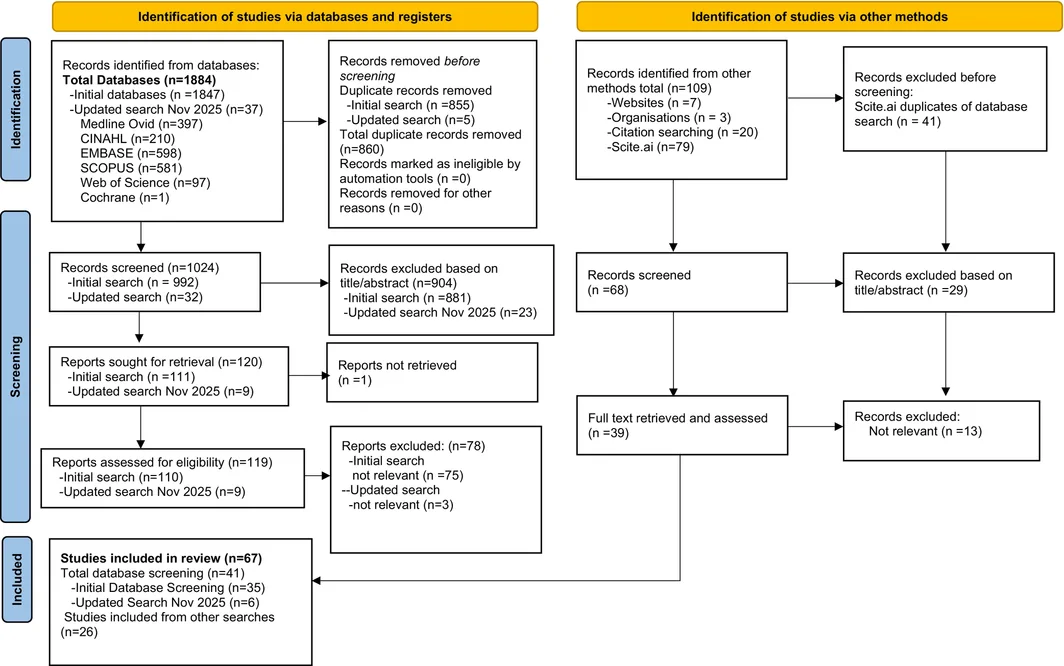
PRISMA ScR flowchart for scoping review (Page et al. 2021).

Database searching identified 1884 records across six databases, comprising 1847 records from the initial search and 37 from an updated search conducted in November 2025. After removing 860 duplicate records, 1024 unique records remained for title and abstract screening. In addition, 109 records were identified through other sources, including Scite.ai (*n* = 79), citation searching (*n* = 20), websites (*n* = 7), and organisations (*n* = 3). Of these, 41 Scite.ai records were duplicates of database records and were removed prior to screening, leaving 68 records for screening from other sources. Title and abstract screening excluded 904 database records and 29 records from other sources. A total of 120 reports were sought for full‐text retrieval; one report could not be retrieved. In total, 119 full‐text reports were assessed for eligibility. Seventy‐eight reports were excluded for failing to meet the inclusion criteria. Sixty‐seven studies met the eligibility criteria and were included in the review. This process is detailed in Figure 1.

#### 2.2.5 Quality Appraisal

Following Joanna Briggs Institute guidance (Peters et al. 2020), a formal quality appraisal was not conducted because this scoping review aimed to map evidence rather than assess study rigour. This approach aligns with the goal of scoping reviews to inform future research and identify knowledge gaps, rather than synthesising outcomes based on methodological strength.

#### 2.2.6 Data Charting and Extraction

The review employed a structured data extraction framework to collect key characteristics, including publication details, population descriptors, and contextual factors. Two independent reviewers piloted the framework on a sample of 10 studies, ensuring reliability through independent charting and consensus discussions to resolve disagreements. Distinctions in hospital settings were clarified. Data were then extracted and charted independently to improve data extraction robustness, with any disagreements resolved through consensus discussion.

#### 2.2.7 Data Abstraction

Data were organised in three Excel spreadsheets based on key variables for consistency and clarity. These included:

- Study characteristics: country, aims, study design, clinical setting and demographics.
- Wearable devices: comparators, make, model, type (e.g., patch, watch), intervention details, measured parameters (HR, RR, SpO_2_, BP, Temperature) and use of artificial intelligence (AI).
- Outcomes: effects on patient length of stay (LOS), transfers, early detection of deterioration, economic impacts, and outcomes for patients and staff.

#### 2.2.8 Data Synthesis

A thematic synthesis was conducted using Braun and Clarke's (2022) six‐phase reflexive thematic analysis framework. Phase 1 involved familiarisation with the data through repeated reading of study findings. In Phase 2, initial codes were generated to capture meaningful features related to clinical, nursing, patient and system‐level outcomes. Coding was primarily inductive, while remaining sensitised to the review objectives and predefined outcome domains. Phase 3 involved developing provisional subthemes by examining patterns of shared meaning across codes. In Phase 4, the dataset was revisited iteratively, using an Excel matrix as an analytic tool to support comparison across studies, refine subthemes, and explore relationships and inconsistencies. Phase 5 involved defining and naming themes through team‐based interpretive discussion, moving from descriptive patterns to higher‐order analytic themes. Phase 6 produced the final narrative synthesis, integrating thematic interpretation with evidence mapping to enhance coherence and transparency. Consistent with Braun and Clarke's (2022) reflexive approach, the analysis was recursive and interpretive.

## 3 Results

### 3.1 Study Characteristics

Sixty‐Seven papers met the inclusion criteria. Table 3 presents the general characteristics of the included papers, summarised in Table 4.

**TABLE 3:** Characteristics of the included studies.

	Author(s)	Year	Title	Country	Aim	Research methodology	Clinical Setting	Participants	Age and Gender
1	Beaman et al.	2023	Potential for remote vital sign monitoring to improve hospital patient sleep: A feasibility study	United States	To assess: (1) patient experience with the vital sign monitoring device, including its impact on sleep; (2) nurse experience caring for patients using the device; (3) the concordance between the device's vital sign measurements and those collected by nurses	Cohort study, conducted prospectively, pilot study	Neurosciences ward	Patients: 21, Nurses: 15	Intervention group: median patient age 42 years (IQR 29, 50), gender 66.7% female. medium nurse age 31, (IQR 28, 44)
2	Becking‐Verhaar et al.	2023	Continuous vital signs monitoring with a wireless device on a general ward: A survey to explore nurses' experiences in a post‐implementation period	Netherlands	To explore nurses' post‐implementation experiences regarding the facilitators and barriers to continuously monitoring patients' vital signs using a wireless device on general hospital wards	Cross‐sectional survey, conducted prospectively	Three general wards: abdominal and oncological surgery, internal medicine and rheumatology, and gastroenterology	Nurses 58, including both vocationally educated and registered nurses	Age: 21–30 years: 72.4%, 31–40 years: 17.2%, 41–50 years: 5.2%, 51–60 years: 5.2% Gender: Male: 15.5%, female: 84.5%
3	Breteler et al.	2020a	Vital signs monitoring with wearable sensors in high‐risk surgical patients. A clinical validation study	Netherlands	To determine whether four different monitoring systems (two wearable patch sensors, a bed‐based system, and a patient‐worn monitor) can reliably measure heart rate and respiratory rate continuously in high‐risk surgical patients	Observational study comparison. conducted prospectively	Surgical step‐down unit	25 high‐risk surgical patients from surgical traumatology or surgical oncology specialties	Age: median 62 years [IQR 51–71] Gender: female: 9 (36%), Male: 16 (64%)
4	Breteler et al.	2020b	Are current wireless monitoring systems capable of detecting adverse events in high‐risk surgical patients? A descriptive study	Netherlands	To describe the ability of currently available wireless sensors to detect adverse events in high‐risk surgical patients by evaluating their capability to detect changes in vital signs prior to and during these events	Observational study conducted prospectively	Surgical step‐down unit and the traumatology or surgical oncology ward	31 high‐risk surgical patients	Age: median 62 years (IQR 20) Gender: 21 male (68%), 10 female (32%)
5	Breteler et al.	2018	Reliability of wireless monitoring using a wearable patch sensor in high‐risk surgical patients at a step‐down unit in the Netherlands: A clinical validation study	Netherlands	To determine whether a wireless ‘patch’ sensor can reliably measure respiratory and heart rates continuously in high‐risk surgical patients, explore its potential as a safety monitor, and verify its robustness in detecting physiological trend patterns before introducing it into clinical practice	Observation comparison study, conducted prospectively, pilot study	Surgical step‐down unit	25 high risk postoperative surgical patients	Age: median 63.0 years (IQR 57.8–71.5) [range 23.0–77.0] Gender: 18 males (72%)
6	Choi et al.	2022	Advantage of vital sign monitoring using a wireless wearable device for predicting septic shock in febrile patients in the emergency department: A machine learning‐based analysis	Korea	To evaluate the predictive performance of a wireless monitoring device that continuously measures heart rate and respiratory rate in febrile but stable patients in the emergency department (ED) using machine learning‐based analysis	A retrospective study, pilot study	Emergency department, specifically in the isolation care unit	468 patients Training set: 277, Validation set: 93, test set: 98 Patients with suspected COVID‐19 The study involved febrile but stable patients	Age: 56.87 ± 18.80 years. Gender: 44.20% male
7	Downey et al.	2019	Reliability of a wearable wireless patch for continuous remote monitoring of vital signs in patients recovering from major surgery: a clinical validation study from the TRaCINg trial	United Kingdom	To validate the accuracy of a wearable remote vital signs monitor in measuring heart rate, respiratory rate, and temperature in postsurgical patients at risk of complications to assess how well the wireless patch system compares to manually recorded measurements in a clinical setting	Single‐centre, feasibility, randomised controlled trial, Conducted prospectively, pilot study	Surgical wards	51 patients who had undergone major elective general surgery	No information on age and gender included in the paper
8	Downey et al.	2018a	Continuous versus intermittent vital signs monitoring using a wearable, wireless patch in patients admitted to surgical wards: Pilot cluster randomised controlled trial	United Kingdom	To evaluate whether continuous remote vital signs monitoring is a practical and acceptable way of monitoring surgical patients, and to use the pilot data to inform an optimise further definitive trial	Cluster‐randomised, parallel‐group, unblinded, controlled, Conducted prospectively, pilot study	Two elective general surgery wards	Surgical patients: total 226 Intervention group 140 Control group 86	Intervention group (Sensium Vitals + intermittent monitoring): Age: mean 65.2 years, range 24–94 years Gender: males: 76 (54.3%), females: 64 (45.7%) Control group (intermittent monitoring alone): age: mean 63.7 years, range 21–92 years Gender: males: 39 (45.4%), females: 47 (54.6%)
9	Downey et al.	2020	Trial of remote continuous versus intermittent NEWS monitoring after major surgery (TRaCINg): A feasibility randomised controlled trial	United Kingdom	To evaluate the feasibility of conducting a large‐scale trial on continuous remote monitoring after major surgery, and to assess the potential safety, efficacy, acceptability, and cost utility of a wearable remote monitoring system compared to standard (National Early Warning System) NEWS monitoring	Randomised, controlled, feasibility trial, conducted prospectively, pilot study	Elective general surgical wards	Patients undergoing elective major abdominal surgery (male and female): 136 total intervention group: 65 control group: 66	Control group (NEWS alone): age: mean 62 years, range 22–87 years gender: males 35 (28.0%), females 30 (24.0%) Intervention group (Sensium Vitals + NEWS): age: mean 65 years, range 36–85 years gender: males 31 (24.8%), females 29 (23.2%)
10	Eddahchouri et al.	2023	The effect of continuous versus periodic vital sign monitoring on disease severity of patients with an unplanned ICU transfer	Netherlands	To compare patient's disease severity upon unplanned ICU transfer before and after the implementation of continuous monitoring (CM), and to assess the effect of CM on ICU and hospital LOS, the need for mechanical ventilation, and ICU mortality	Retrospective before‐and‐after study	Surgical wards. The study focused on unplanned intensive care unit transfers from these wards	Patients primarily having upper gastrointestinal‐and/or hepato‐pancreatic‐biliary cancer, and internal medicine wards with general internal medicine, rheumatology, and infectious disease patients: 136 unique patients with unplanned ICU transfers; Pre‐CM period: 82 patients with 93 ICU transfers; post‐CM period: 54 patients with 59 ICU transfers	Control group: Age: median 67 years (IQR 58–74) Gender: female 41% Intervention group: age median 64 years (IQR 55–71) Gender: female—46%
11	Ghiasi et al.	2022	Sepsis mortality prediction using wearable monitoring in low‐middle income countries	Vietnam	(1) To assess the feasibility of using wearable sensors instead of traditional bedside monitors in the management of sepsis care in hospital‐admitted patients (2) To introduce automated prediction models for mortality prediction in sepsis patients (3) To investigate the potential of heart rate variable measures obtained from wearable sensors for automatic prediction of in‐hospital mortality at early admission stages. (4) To propose an interpretable automatic machine learning based solution for long‐term prediction of hospital discharge outcomes in critically ill sepsis patients using data collected during the first day of admission	Prospective study	Tertiary referral centre for infectious diseases	50 sepsis patients were initially recruited, with 40 included in the final analysis	Age (≥ 64): 12 overall, 2 in the death group, 10 in the survival group gender: male: 67.5% overall, 64.3% in the death group, 72% in the survival group Age (50–64): 7 overall, 1 in the death group, 6 in the survival group Age (< 50): 21 overall, 11 in the death group, 10 in the survival group
12	Haahr‐Raunkjaer et al.	2022b	Continuous monitoring of vital sign abnormalities; association to clinical complications in 500 postoperative patients	Denmark	To assess the association between abnormal vital signs, inspired by Early Warning Score thresholds, and subsequent serious adverse events (SAEs) in patients undergoing major abdominal surgery. The researchers aimed to explore if severe abnormal vital signs were associated with the development of SAEs and hypothesised that these abnormalities would be more prolonged and frequent in patients with SAEs	Observational cohort study	Surgical ward	491 postoperative patients undergoing major abdominal cancer surgery	Age: median 70 years gender: 63% male, 37% female
13	Haveman et al.	2021	Continuous monitoring of vital signs with the Everion biosensor on the surgical ward: a clinical validation study	Netherlands	To assess the validity and reliability of the Everion biosensor in continuously monitoring vital signs on a surgical ward by comparing its measurements with those taken by nurses. The study also aimed to investigate the feasibility and quality of data collected through continuous telemonitoring in a clinical setting	Blinded observational study, conducted prospectively, pilot study	Surgical wards	19 surgical patients scheduled for elective open abdominal surgery	Age: median 68 years (IQR 62.5–72.5). gender: male 73.7%, female 26.3%
14	Helmer et al.	2025	The use of wearable sensor technology to enhance supportive care in hospitalised patients (Support trial): A prospective preliminary pilot study	Germany	(1) To evaluate the feasibility of continuous vital sign monitoring in hospitalised palliative care patients using wrist‐worn and chest‐wall devices. (2) To determine the time interval between the signal loss of the wrist monitor and the documented time of death. (3) To assess whether continuous vital sign monitoring is feasible with wrist and chest monitors in palliative care patients. (4) To evaluate the completeness of the acquired datasets	Prospective pilot study	Palliative care	275 patients were screened, with 7 patients ultimately participating in the study	Age range 36–83 years, IQR 55.5; 75.5 Gender: 57.1% male
15	Helmer et al.	2024	Reliability of continuous vital sign monitoring in post‐operative patients employing consumer‐grade fitness trackers: A randomised pilot trial	Germany	To evaluate the accuracy and reliability of heart rate, blood oxygen saturation (SpO_2_), and respiratory rate measurements by consumer‐grade fitness trackers compared to clinical standards	Observational, randomised conducted prospectively, pilot study	Post‐anaesthesia care units and, if necessary, monitoring continued in intermediate care or intensive care unit	Post‐operative patients 36 initially included, 26 analysed Apple: 10 Garmin: 12 Withings: 4	Age: mean 65.5 years (IQR 12.25). Gender: male: 14 (53.85%), female: 12 (46.15%)
16	Helmer et al.	2022	Accuracy and systematic biases of heart rate measurements by consumer‐grade fitness trackers in postoperative patients: Conducted prospectively clinical trial	Germany	To evaluate the accuracy of heart rate measurements by consumer‐grade fitness trackers (Apple Watch 7, Garmin Fenix 6 Pro, Withings ScanWatch, and Fitbit Sense) against the clinical gold standard (electrocardiography) in postoperative patients. The researchers wanted to find out if these devices could be reliably used in a medical setting for monitoring heart rate	Validation study, conducted prospectively	Postanaesthetic care unit	112 non‐sedated postoperative patients who had undergone moderate to major surgery	Age: median 68 years. gender: male, 62.5% female, 37.5%
17	Hernandez‐Silveira et al.	2015	Assessment of the feasibility of an ultra‐low power, wireless digital patch for the continuous ambulatory monitoring of vital signs	United Kingdom	To assess the agreement between a new ultra‐low power, wireless digital patch for continuous ambulatory monitoring of vital signs and a widely used clinical vital signs monitor To evaluate the system's ability to automatically identify and reject invalid physiological data, thereby minimising false alarms and erroneous reporting To evaluate the performance of the Sensium Vitals digital patch in a hospital environment	Feasibility trial, conducted prospectively, pilot study	Operating room and general medical ward	Patients: 61 total 20 undergoing elective surgery, 41 with comorbid conditions	Control group (Group 1): mean age 49 years (SD ±16), 13 males 7 female participants. Intervention group (group 2):Low voltage/variable QRS morphology: mean age 62 years (SD ±12), 7 male, 2 female participants Atrial fibrillation: mean age 78 years (SD ±6), 6 males, 4 female participants High BMI: mean age 51 years (SD ±11), 4 males, 6 Female participants 16 Diabetes: mean age 51 years (SD ±11), 9 male, 9 female
18	Iqbal et al.	2022	Outcomes of vital sign monitoring of an acute surgical cohort with wearable sensors and digital alerting systems: A pragmatically designed cohort study and propensity‐matched analysis	United Kingdom	To test a wearable sensor and alerting system and its influence on clinical outcomes in an acute surgical cohort, with a focus on understanding the implementation experience	Pre‐post implementation trial, conducted prospectively	Acute surgical unit	Patients with common surgical presentations such as acute abdominal pain and bleeding symptoms, referred from the emergency department or primary care. patients: Pre‐implementation group: 141 Post‐implementation group: 141	Pre‐implementation Age 51 (35–66) Male 125 (45%) Female 54 (55%) Post implementation = 141 age 55 (36–73) Male 74 (52%) Female 67 (48%) After propensity score matching Pre‐implementation: 141 Age 51 (39–71) Male 69 (49%) Female 72 (51%) Post implementation 141 Age 55(36–73) Male 74 (52%) Female 67 (48%)
19	Ishikawa et al.	2021	Patient SafetyNet for the evaluation of postoperative respiratory status by nurses: A presurvey and postsurvey study	Japan	To assess the effect of the patient safety net system on post operative respiratory evaluation by nurses in general wards	Pre‐post implementation survey, conducted prospectively	General medical and surgical wards	Nurses 75	Years of experience > 11 years 16 nurses (21.3%), 6–10 years 29 (38.7%) of nurses < 5 years in 30 (40%) of nurses
20	Itelman et al.	2022	Assessing the usability of a novel wearable remote patient monitoring device for the early detection of in‐hospital patient deterioration: observational study	Israel	To assess the potential of a novel wearable remote patient monitoring (RPM) device to provide timely alerts in patients at high risk for deterioration and to evaluate whether frequent RPM can detect early signs of deterioration using grouped signal‐based scores compared to traditional clinical detection methods	Prospective observational study with retrospective analysis	Two general wards	217 patients determined at high risk to deteriorate on admission and assigned to a telemetry bed	Median age: 71 years (IQR 62–78) Gender: 59.9% male
21	Jacobs et al.	2021	Reliability of heart rate and respiration rate measurements with a wireless accelerometer in post bariatric recovery	Netherlands	To assess the reliability and determine the accuracy of the Healthdot device in measuring heart rate and respiration rate compared to the gold standard patient monitor, specifically in bariatric patients during their postoperative period in a post‐anaesthesia care unit	Comparative study, conducted prospectively	Post anaesthesia care unit	26 patients (post‐bariatric surgery)	Age: 46.5 years (median) Gender: 10 male 16 female
22	Jacobsen et al.	2022	Feasibility of wearable‐based remote monitoring in patients during intensive treatment for aggressive hematologic malignancies	Germany	To assess the feasibility of using a wearable device for continuous monitoring of vital signs and physical activity in patients undergoing intensive treatment for aggressive hematologic malignancies, to evaluate patient adherence to the wearable despite high symptom burden, and to explore the potential for improved early detection and treatment of complications through wearable‐based remote patient monitoring	A single‐arm, single‐centre observational trial with retrospective data review, pilot study	Department of haematology, oncology, and clinical immunology. It involved both inpatient and outpatient settings	Patients: 79 total 54 inpatients, 25 outpatients undergoing treatment for hematologic malignancies	Inpatient cohort: Age: median 56 years (IQR 49–62) Gender: male 55.6%, female 44.4% Outpatient cohort: Age: median 54 years (IQR 50–62) Gender: male 56.0%, female 44.0%
23	Jokinen et al.	2022	Wireless single‐lead ECG monitoring to detect new‐onset postoperative atrial fibrillation in patients after major noncardiac surgery: A conducted prospectively observational study	Denmark	To describe the frequency of new‐onset postoperative atrial fibrillation (POAF) in patients undergoing major gastrointestinal cancer surgery using wireless monitoring, and to investigate the association between preceding vital sign deviations and the occurrence of POAF. The researchers wanted to find out if wireless single‐lead ECG monitoring could detect POAF more frequently compared to routine monitoring methods and if there were any significant associations between deviating vital signs and the subsequent development of POAF	Observational cohort study, conducted prospectively	Surgical wards	398 patients undergoing major gastrointestinal cancer surgery, with no intervention or control groups as it is an observational study	Age: patients ≥ 60 years; median age Post‐operative atrial fibrillation: 74 years, no post‐operative atrial fibrillation: 70 years Gender: post‐operative atrial fibrillation: 58% male
24	Joshi, et al.	2025	Performance of Continuous Digital Monitoring of Vital Signs with a Wearable Sensor in Acute Hospital Settings	United Kingdom	To evaluate the reliability of a wearable sensor for monitoring heart rate, respiratory rate, and temperature in acutely unwell hospital patients. To compare the performance of the wearable sensor with standard nurse observations. To define acceptable discrepancies between nurse and device readings, particularly for respiratory rate	Cohort study, conducted prospectively	Acute medical and surgical wards	500 patients who were acutely unwell and admitted to a hospital	Age: 18–95 years gender: Not mentioned
25	Joshi et al.	2022a	A pilot study to investigate real‐time digital alerting from wearable sensors in surgical patients	United Kingdom	To investigate if real‐time digital alerts sent to healthcare staff can improve the time taken to identify unwell patients and those with sepsis To assess the use of continuous monitoring through wearable sensors, to evaluate the time to alert acknowledgment, and to determine whether patients with deterioration were identified and treated To assess the feasibility of using these technologies in a hospital ward setting before wider implementation	Cohort study conducted prospectively, pilot study	Surgical assessment unit	50 patients acutely unwell surgical patients The study also involved nurses, but their exact number is not specified	No specific information on age range or gender of participants
26	Joshi et al.	2022b	Perceptions on the use of wearable sensors and continuous monitoring in surgical patients: interview study among surgical staff	United Kingdom	To understand the views of surgical team members regarding novel wearable sensors for surgical patients, conduct a comprehensive exploration of interdisciplinary staff views on wearable sensor technology, and assess the acceptability of wearable sensors among those who use them	Qualitative study using semi structured interviews and thematic analysis, conducted prospectively	Surgical wards with acutely unwell surgical patients	Senior and junior surgeons, senior and junior nurses: 48 participants in total	No information on age and gender of participants
27	Joshi et al.	2021	Short‐term wearable sensors for in‐hospital medical and surgical patients: mixed methods analysis of patient perspectives	United Kingdom	To explore patient experiences with wearable sensor technology	Mixed methods, combining quantitative (questionnaire) and qualitative (semi‐structured interviews), conducted prospectively	Medical and Surgical wards	Acutely unwell patients: 500 total; 453 completed the questionnaire. 12 surgical patients participated in interviews	Questionnaire participants: mean age 57 years Gender 51% female Interview participants: mean age 49 years, 50% male
28	Kachel et al.	2021	A pilot study of blood pressure monitoring after cardiac surgery using a wearable, non‐invasive sensor	Israel	To evaluate the accuracy of continuous blood pressure measurements obtained from a non‐invasive, wireless photoplethysmography (PPG)‐based device using two configurations wristwatch and chest‐patch monitors compared to an arterial line in post‐cardiac surgery patients	Comparative clinical trial, conducted prospectively, pilot study.	Cardiac surgery and intensive care unit	10 patients post‐cardiac surgery	Subject 1: male, 60 years Subject 2: female, 75 years Subject 3: female, 45 years Subject 4: female, 45 years Subject 5: male, 60 years Subject 6: male, 81 years Subject 7: male, 35 years Subject 8: female, 76 years Subject 9: male, 64 years Subject 10: male, 57 years
29	Kooij et al.	2022	Remote continuous monitoring with wireless wearable sensors in clinical practice, nurses' perspectives on factors affecting implementation: A qualitative study	Netherlands	To evaluate factors affecting the implementation of continuous monitoring using wireless wearable sensors by assessing nurses' experiences on the nursing ward To explore nurses' expectations for the use of continuous monitoring in the home setting	Qualitative study using semi‐structured interviews, conducted prospectively; related to pilot implementations but not explicitly a pilot study itself	Bariatric patients post‐surgery, heart or heart valve surgery patients, clinically unstable patients, vulnerable elderly patients, and patients with pulmonary, neurological, gastrointestinal and liver diseases	16 nurses	Age: mean 34.1 years (SD 11.2), range 22–56 years Gender: 100% women
30	Kroll et al.	2016	Accuracy of a wrist‐worn wearable device for monitoring heart rates in hospital inpatients: A prospective observational study	Canada	To evaluate the accuracy of heart rate monitoring by a personal fitness tracker among hospital inpatients, specifically comparing it to continuous ECG monitoring and pulse oximetry to assess its potential use in clinical settings	Observational study, conducted prospectively	Intensive care unit	50 stable patients Including medical, surgical, trauma, and neurosciences patients. The study focused on stable ICU patients not receiving mechanical ventilation, continuous analgesia, or sedation	Age: mean 64 years. Gender: male: 26 (52%), female: 24 (48%)
31	Leenen et al.	2024	Impact of wearable wireless continuous vital sign monitoring in abdominal surgical patients: Before and after study	Netherlands	To explore the effect of continuous monitoring of vital signs on clinical outcomes in major abdominal surgery patients, with a primary focus on the length of stay and secondary aims to assess a broad range of other clinical outcome measures	A single‐centre before‐after study, both retrospective and conducted prospectively	General surgery ward	Patients: 908 control group: 714, intervention group: 194 focusing on patients undergoing elective major abdominal surgeries, specifically colorectal and hepatopancreatobiliary surgeries	Control group: age: mean age 66.8 years gender: 50.6% male, 49.4% female Intervention group: age: mean age 68.1 years Gender: 54.6% male, 45.4% female
32	Leenen et al.	2023	Process evaluation of a wireless wearable continuous vital signs monitoring intervention in 2 general hospital wards: A mixed methods study	Netherlands	To assess and compare intervention fidelity of wireless wearable continuous vital signs monitoring in two hospital wards To evaluate various aspects of the implementation process such as implementation fidelity, technical fidelity, perceived appropriateness, acceptability, usability, adoption, and feasibility according to nurses	A mixed methods sequential explanatory design, conducted prospectively	Two wards: A surgical and medical ward (focusing on gastrointestinal and vascular surgery patients) An internal medicine ward (including general internal medicine and gastroenterology patients)	Patients: 358 Surgical ward: 248, internal gastrointestinal and vascular surgery patients Medicine ward: 110. Internal medicine and gastroenterology patients Nurses: 148 Surgical ward: 71 Internal medicine ward: 77	Surgical ward patients: age: 67.8 years (mean) gender: 55.6% male Internal medicine ward: age: 71.2 years (mean) gender: 70% male
33	Leenen et al.	2022a	Feasibility of wireless continuous monitoring of vital signs without using alarms on a general surgical ward: A mixed methods study	Netherlands	To determine the feasibility of continuous vital sign monitoring without using alarms, focusing on interval trend monitoring. The objectives included assessing feasibility in terms of acceptability by healthcare professionals and fidelity of the monitoring system on a general surgical ward	An explanatory sequential mixed methods study, conducted prospectively.	Abdominal surgical ward	Patients: 56. Involving patients scheduled for elective colorectal, hepatic, or pancreatic resections Healthcare professionals: 46 (including nurses and doctors)	Patients: Age median 71 years (IQR 63–80), gender male: 30, female: 26 Healthcare professionals: age median 28 years (IQR 24.5–41.3) Gender: male: 3 female: 42
34	Leenen et al.	2022b	Nurses' experiences with continuous vital sign monitoring on the general surgical ward: A qualitative study based on the behaviour change wheel	Netherlands	To provide insight into the capability, opportunity, and motivation of nurses working with continuous vital signs monitoring systems, to inform and support future implementation efforts using the behaviour change wheel	Qualitative, study using semi‐structured interviews and thematic analysis, conducted prospectively	Surgical ward	12 nurses	Age median of 27.5 years (IQR 23–31.5). All participants were female
35	Leenen et al.	2021	Feasibility of continuous monitoring of vital signs in surgical patients on a general ward: An observational cohort study	Netherlands	To determine the feasibility, in terms of acceptability and system fidelity, of continuous vital signs monitoring with the Sensium Vitals device among abdominal surgery patients on a general surgery ward	Observational cohort study, conducted prospectively, pilot study	Surgical ward	Patients: 30. Focusing on postoperative abdominal surgical patients undergoing elective colorectal or pancreatic resection Nurses: 23	Intervention group: age mean: 66 years (SD 10) Male: 17 Female: 13
36	Li et al.	2019	A pilot study of respiratory rate derived from a wearable biosensor compared with capnography in emergency department patients	United States	To compare respiratory rates derived from a Philips wearable biosensor with those derived from capnography, to assess if the biosensor could provide comparable measurements in emergency department patients with respiratory complaints	Conducted prospectively, pilot study	Emergency department	Patients: 17 Involving adult patients with respiratory complaints	Age mean: 61 years Gender: 59% male
37	Liu et al.	2020	Evaluation of a wearable wireless device with artificial intelligence, iThermonitor WT705, for continuous temperature monitoring for patients in surgical wards: A prospective comparative study	China	To evaluate the performance and feasibility of the iThermonitor WT705, a new‐generation, non‐invasive, wireless axillary thermometer with AI, for continuous temperature monitoring in surgical patients during the perioperative period. The researchers wanted to determine its accuracy, precision, and potential for practical application in clinical settings	Observational study, conducted prospectively	Biliary surgery, operating room and the post anaesthesia care unit	526 adult surgical patients	Total participants: 526 Average age: 53.47 years (range: 15–86 years) Gender: male: 197 (37.5%), female: 329 (62.5%)
38	Lockhorst et al.	2025	Monitoring vital signs with continuous monitoring after major gastrointestinal surgical procedures: The patient, nurse and physician perspective	Netherlands	To evaluate user satisfaction of a new wireless monitoring system measuring vital signs continuously, from the perspectives of patients, nurses, and physicians	Qualitative study, conducted prospectively	Surgical ward	Patients: 198Nurses: 88Physicians: 19	Not mentioned
39	Logaras et al.	2022	Risk assessment of COVID‐19 cases in emergency departments and clinics with the use of real‐world data and artificial intelligence: Observational study	Greece	To develop a smart system for dynamically prioritising COVID‐19 patients through continuous monitoring of vital signs using a wearable biosensor device, and to estimate the likelihood of deterioration of each case using AI models. The study aimed to create a clinical decision support system to assist in patient management and improve resource management in healthcare facilities	Observational cohort study, conducted prospectively	Emergency department and COVID‐19 inpatient unit	157 adult patients diagnosed with COVID‐19 from the emergency department and COVID‐19 inpatient unit, aged ≥ 18 years	Age: mean 52.8 years (SD 19.6) Gender: 56.1% male
40	McGrath et al.	2019	Improving patient safety and clinician workflow in the general care setting with enhanced surveillance monitoring	Lebanon	To evaluate the impact of a wireless patient monitoring system on clinician workflow and patient care, with a specific focus on early detection of patient deterioration to reduce failure‐to‐rescue events in general care hospital units	Observational studies, pre and post implementation., conducted prospectively	2 surgical wards, 2 medical wards	The study included 71 beds across two surgical units where the enhanced surveillance monitoring system was implemented. The study also involved control units (three units, 61 beds total) where the surveillance system remained unchanged	The paper does not provide a breakdown of the number of individual participants, gender distribution, or specific age details
41	Murali et al.	2020	Heart rate and oxygen saturation monitoring with a new wearable wireless device in the intensive care unit: Pilot comparison trail	Switzerland	To test the clinical value and usefulness of the Smart Cardia wireless sensor device in monitoring heart rate (HR) and oxygen saturation (SpO_2_) in ICU patients To assess the safety and tolerance of the device and evaluate the correlation of HR and SpO_2_ measurements obtained by the Smart Cardia device with those obtained by a conventional intensive care unit‐grade monitoring system The study was primarily designed to validate the sensor accuracy of the Smart Cardia system	Observational study, conducted prospectively, pilot study	Cardiac intensive care unit	58 ICU patients with cardiovascular conditions, primarily post‐cardiac surgery or with stable hemodynamic conditions post‐myocardial infarction	Total participants: 58 male: 42 Mean age: 70 years (SD 12) Female: 16, mean age: 74 years (SD 8)
42	Nantume et al. – child study needs removing	2022	Accuracy and reliability of a wireless vital signs monitor for hospitalised patients in a low resource setting	Uganda	To evaluate the accuracy and reliability of the neoGuard device compared to a conventional bedside monitor in a low‐resource setting, measure the level of agreement between the neoGuard technology and the Edan iM8 monitor, and assess the reliability and feasibility of using a wearable sensor on the patient's forehead	Single‐arm comparison study, conducted prospectively, pilot study	Recovery ward	30 low‐acuity patients in the recovery ward	Male: median age 49 (IQR 35–53), 10 participants Female: median age 27 (IQR 23–32), 20 participants
43	Page et al.	2025	Continuous vital sign monitoring of acute Lassa fever using wearable biosensor devices in West Africa Check for updates	Sierra Leone	To use wearable biosensor devices to remotely monitor hospitalised individuals with acute Lassa fever To describe vital sign trends associated with clinical outcomes To evaluate the feasibility and efficacy of this approach in a resource‐limited setting To identify individuals at increased risk for poor outcomes by detecting age‐specific tachycardia and reductions in heart rate variability To assess technical challenges and limitations of using wearable biosensors in resource‐limited settings	Observational study, conducted prospectively	Lassa ward with enhanced biocontainment measures	There were initially 17 participants, but only 8 were included in the final analysis due to data quality issues	Median age: 6.5 years (range: 0.4–40 years) Gender: 50% female
44	Patel et al.	2022	Perceptions of patients and nurses regarding the use of wearables in inpatient settings: A mixed methods study	Canada	To highlight the key barriers and facilitators involved in adopting wearable technology in acute care settings using patient and clinician feedback	Mixed methods, feasibility study	General internal medicine units, hospitals	39 patients, 30 nurses	Patients age median 71.7. years Gender: male 16 (41%) female 23 (59%)
45	Peelen et al.	2025	Exploring the Relationship Between Continuously Monitored Vital Signs, Clinical Deterioration, and Clinical Actions	Netherlands	To Correlate clinical actions with clinical endpoints and deviating vital signs. To Determine the relationships between clinical actions and clinical deterioration. Test two hypotheses: 1. Correlation between clinical actions and negative clinical endpoints/admission duration. 2. Correlation between clinical actions and alarming minutes during vital sign monitoring. Explore clinical actions as an intermediate outcome measurement for clinical deterioration	Cohort study, conducted prospectively	Gastro‐ intestinal oncological surgery ward an internal medicine ward	Number of participants: 1529 total, with 68 having negative clinical endpoints	Age—60.1 ± 25.1 years Gender: male—51.9%, female—48.1%
46	Pietrantonio et al.	2019	Technological challenges set up by continuous wireless monitoring designed to improve management of critically ill patients in an internal medicine unit (LIMS study): Study design and preliminary results	Italy	To assess the efficacy of a wireless continuous monitoring system in reducing major complications, improving outcomes and quality of care in critically ill patients, and reducing hospitalisation costs compared to traditional monitoring. The study also aimed to evaluate the management of these patients using wireless monitoring in the first 72 h of hospitalisation	Randomised controlled open‐label multi‐centre study, conducted prospectively, pilot study	Internal medicine ward	All patients with a MEWS greater than or equal to 3 and/or greater than or equal to 5 at admission regardless of the reason for hospitalisation and all patients with poor glycaemic control and/or severe fluid and electrolyte balance were deemed eligible for continuous monitoring using the WIN@Hospital system In the first 12 months, 95 patients were enrolled, with 89 evaluable	Age mean: 80.5 years. Male: 35 (39.3%), Female: 54 (60.7%). Experimental arm 43 Male: 15 (34.9%) Female: 28 (65.1%) Control arm 46 male, 20 (43.5%) Female,26 (56.5%) female
47	Posthuma et al.	2020	Remote wireless vital signs monitoring on the ward for early detection of deteriorating patients: A case series	France, The Netherlands, United Kingdom	To illustrate how remote wireless monitoring systems might contribute to improved patient surveillance and to demonstrate their potential to reduce the time to detect deteriorating patients on medical and surgical wards	Case series	Five hospitals across three European countries, specifically in medical and surgical wards, as well as an emergency department	Patients: 9	Case 1: male, 78 years Case 2: male, 58 years Case 3: male, 73 years Case 4: male, 81 years Case 5: male, 75 years Case 6: female, 78 years Case 7: female, 87 years Case 8: male, 53 years Case 9: male, 85 years
48	Reed et al.	2018	Detection of physiological deterioration by the SNAP40 wearable device compared to standard monitoring devices in the emergency department: The SNAP40‐ED study	United Kingdom	To compare the ability of a prototype ambulatory monitoring device to detect deteriorations in vital sign physiology of ED patients earlier than standard monitoring techniques. Secondary aims included measuring the time ED staff spent on observations and alarms, assessing clinical escalation or de‐escalation due to changes in vital signs, and studying patient and staff experiences with the novel versus standard monitoring techniques	Single‐centre, open‐label, observational cohort study conducted prospectively. It was not, a pilot study itself but followed a small pilot study	Emergency department	250 patients in the Emergency Department with high/medium acuity (Manchester triage category 2 and 3), aged over 16 years Of these, 238 had cognitive capacity 51 patients underwent wired monitoring, while the remainder had their vital signs recorded manually	Age: no specific median or mean age provided Gender: Male: 133 (53.2%) Female: 117 (46.8%)
49	Restrepo et al.	2021	Remote monitoring of critically ill post‐surgical patients: Lessons from a biosensor implementation trial	United States	To study the implementation of biosensors in a clinical setting, compare the accuracy of biosensor data with standard clinical monitoring, assess the acceptance of biosensor technology by patients, providers, and nurses, and describe the experiences and challenges during the implementation process in an ICU setting	Observational study, conducted prospectively, pilot study	Intensive care unit	Patients: 8; critically ill post‐surgical patients providers taking care of patients: 13 (including MDs: 8, & RNs: 5) Providers wearing devices: 16	Patients: age 59 ± 9 years Gender: 2 males, 6 females
50	Sharabi et al.	2023	Assessing the use of a non‐invasive monitoring system providing cardio‐pulmonary parameters following revascularization in STEMI patients	Israel	To assess the use of a novel non‐invasive monitor providing multiple cardio‐pulmonary parameters in ST‐segment elevation myocardial infarction (STEMI) patients after primary percutaneous coronary intervention (PPCI)	Observational prospective cohort study, retrospective analysis	Intensive cardiac care unit	Patients 15 Nurses 12	Patients: age 41–72 median 52.8 years. Gender: Males 14 (93.3%) Female 1 (6.7%).
51	Steinhubl et al.	2016	Validation of a portable, deployable system for continuous vital sign monitoring using a multiparametric wearable sensor and personalised analytics in an Ebola treatment centre	Sierra Leone	To validate a portable, deployable system for continuous vital sign monitoring using a wireless, wearable sensor and personalised analytics platform, to improve patient care and healthcare worker safety in low‐resource settings, and to demonstrate its feasibility and potential benefits in any healthcare setting worldwide	Proof of concept study, conducted prospectively, pilot study	Ebola treatment centre	Patients: 26 suspected or confirmed to have Ebola virus disease	Age: median 32.5 years (range 20–70 years) Gender: 65% male 35% female
52	Stellpflug et al.	2021	Continuous physiological monitoring improves patient outcomes	United States	To improve early recognition of patient deterioration and timely intervention using digital continuous vital sign monitoring technology, enhance patient surveillance, and measure success through metrics such as rapid response team activation trends, intensive care unit length of stay, early identification of deterioration, and nursing satisfaction	Quality improvement project, conducted prospectively, pilot study	General medical unit	Patients: 547 nurses: 58 RNs surveyed	No information on age and gender of participants is included in the paper
53	Tenge et al.	2025	Wireless patches for continuous vital sign monitoring, symptoms and medication at the end‐of‐life in the palliative care unit: A prospective observational study	Germany	To assess the feasibility of continuous vital sign monitoring using wearable patches in the palliative care unit. To analyse vital sign changes at the end‐of‐life and their correlation with symptoms and medication use to enhance clinical prognostication, symptom detection, and management	Observational study, conducted prospectively	Palliative care unit	30 patients included in the final analysis	Age median: 70 years (IQR: 61–81 years)male, 46.7% female 53.3%
54	Totuk et al.	2023	Reliability of smartphone measurements of peripheral oxygen saturation and heart rate in hypotensive patients: Measurement of vital signs with smartphones	Turkey	To investigate the accuracy rate of smartphones in measuring peripheral oxygen saturation and heart rate in hypotensive patients and to determine whether smartphones could be reliably used for these measurements	Observational study, conducted prospectively	Emergency department	200 patients with conditions such as asthma, COPD, congestive heart failure, pneumonia, sepsis, cerebrovascular disease, and multiple trauma, received a diagnosis of hypertension and required an arterial blood gas analysis	Age mean: 59.02 years (SD: 17.47) Gender: male: 41.5% female: 58.5%
55	Turan et al.	2019	Incidence, severity and detection of blood pressure perturbations after abdominal surgery: A prospective blinded observational study	United States	To evaluate the incidence and severity of postoperative hypotension and hypertension in adults recovering from abdominal surgery, and to determine the extent to which these serious perturbations were missed by routine vital‐sign assessments. The study also aimed to assess whether non‐invasive continuous blood pressure monitoring could detect more instances of hypotension and hypertension compared to routine assessments	Observational, comparative study, conducted prospectively	Surgical wards	312 adult patients aged who underwent abdominal surgery lasting at least 2 h under general anaesthesia They were part of two clinical trials: one evaluating intravenous acetaminophen and another comparing pain management techniques	Age: 49 ± 15 years Gender: male: 155 (50%), female: 157 (50%)
56	Van Der Stam et al.	2023a	A wearable patch based remote early warning score (REWS) in major abdominal cancer surgery patients	Netherlands	To develop a REWS system using continuous measurements from a wearable device and to compare its diagnostic performance for detecting deterioration with the conventional modified early warning score (MEWS)	Single‐centre trial, conducted prospectively	Surgical wards	Patients: 103 All underwent major abdominal cancer surgery	Age: median 64 years (IQR 57–70). Gender: 45% female
57	Van Der Stam et al.	2023b	The accuracy of wrist‐worn photoplethysmogram‐measured heart and respiratory rates in abdominal surgery patients: Observational prospective clinical validation study	Netherlands	To assess the accuracy of heart rate and respiratory rate measurements obtained via a wearable photoplethysmography wristband in postoperative patients	Observational clinical validation study, conducted prospectively	Postanaesthetic care unit and intensive care unit	Post‐abdominal surgery patients: 68 initially enrolled, 62 included for HR analysis, 54 included for RR analysis	Age: mean 55 years (SD 15). Gender: 33 females (53%)
58	Van Goor et al.	2021	Can continuous remote vital sign monitoring reduce the number of room visits to patients suspected of COVID‐19: A quasi‐experimental study	Netherlands	To assess the impact of continuous remote vital sign monitoring on the number of patient room visits, the number of visits primarily for obtaining vital signs and the amount of personal protection equipment used for patients suspected of COVID‐19 during the SARS‐CoV‐2 pandemic	Observational before‐after study conducted prospectively	General ward with private rooms designated for patients suspected of COVID‐19	Patients: 209 individuals suspected of having COVID‐19. Nurses: number not specified but involved in care and monitoring	No information on age and gender
59	van Nort et al.	2024	Three years of continuous vital signs monitoring on the general surgical ward: Is it sustainable? A qualitative study	Netherlands	To gain insight into’ and patients' perspectives regarding the use and innovation of a continuous vital signs monitoring system, 3 years after its introduction, and to explore how it can be further innovated to ensure sustainable and effective use	Qualitative study, conducted prospectively	Gastrointestinal surgical oncology unit	Key‐user nurses: 4 Regular nurses: 6 Five patients recovering from major abdominal surgical procedures related to oncology	Age: no data provided Gender Key user nurses: 1 male, 3 female regular nurses all female patients: 3 male, 2 female
60	Van Velthoven et al.	2023	ChroniSense national early warning score study: Comparison study of a wearable wrist device to measure vital signs in patients who are hospitalised	United Kingdom	To evaluate the clinical validity of a semiautomated wearable wrist device (ChroniSense Polso) for measuring vital signs and providing NEWS in hospitalised patients. The primary objective was to assess the agreement between the wearable device's measurements and standard manual measurements for respiratory rate, oxygen saturation, body temperature, systolic blood pressure, and heart rate Secondary objectives included evaluating the agreement on the total NEWS, incidence of adverse events, and user acceptance of the device	Validation study	Cardiac care unit	132 patients with cardiac conditions such as acute coronary syndrome, chronic heart failure, and arrhythmia. They were adult patients requiring vital sign measurements at least every 6 h	Age mean: 62 years (SD 15.81) gender: 77.3% male (102/132)
61	Vroman et al.	2023	Continuous vital sign monitoring in patients after elective abdominal surgery: A retrospective study on clinical outcomes and costs	Netherlands	To assess changes in clinical outcomes and in‐hospital costs upon the implementation of continuous vital sign monitoring using a wearable biosensor in postsurgical patients on a general ward. The researchers wanted to find out if this intervention could lead to fewer intensive care unit admissions, shorter hospital stays, and reduced costs	A retrospective pre‐post observational study.	General surgical ward	Patients: 855 total Control group: 651 Intervention group: 204 Postoperative gynaecology and intestinal patients who qualify for the enhanced recovery after surgery protocol	Control group: age mean, 66.7 years male = 49.9% Intervention group: age mean 65.3 years male 56.9%
62	Watkinson et al.	2020	Early detection of physiological deterioration in post‐surgical patients using wearable technology combined with an integrated monitoring system: A pre‐and post‐interventional study	United Kingdom	To assess whether ambulatory (wearable) physiological monitoring combined with a system that continuously merges physiological variables into a single “risk” score (VSI) changes care and outcomes in patients after major surgery. The objective was to evaluate the efficacy of this combined monitoring approach in improving early detection of physiological deterioration in post‐surgical patients	Prospective, before‐and‐after interventional study It can be considered a pilot study as it was the first to link real‐time risk score estimation with ambulatory monitoring	Upper‐gastrointestinal surgical ward	Patients undergoing major upper‐gastrointestinal surgery There were 200 participants in phase I and 207 participants in phase II	Phase I: median age: 63 years (IQR: 54–70) Male: 111 (55.5%) Female: 89 (44.5%) Phase II: median Age: 63 years (IQR: 51–69) Male: 121 (58.5%) Female: 86 (41.5%)
63	Weenk et al.	2020	Continuous monitoring of vital signs in the general ward using wearable devices: Randomised controlled trial	Netherlands	To identify positive and negative effects, barriers, and facilitators for the use of two wearable devices (ViSi Mobile and HealthPatch) and to provide an overview of the experiences and expectations of patients, relatives, nurses, physician assistants, and medical doctors regarding continuous monitoring with these devices in the general ward	Randomised controlled trial, conducted prospectively	Internal medicine and surgical wards	Patients: 90 30 in ViSi Mobile group 30 in HealthPatch group, 30 in control group Nurses: 20 Physician assistants: 3 Medical doctors: 6	ViSi Mobile: age: 63 years (range 26–76) Gender: male: 18 (60%), female: 12 (40%) HealthPatch: age: 56 years (range 27–88) Gender: male: 22 (73%), female: 8 (27%) Control group: age: 62 years (range 34–77) Gender: male: 20 (67%), female: 10 (33%)
64	Weenk et al.	2019	Wireless and continuous monitoring of vital signs in patients at the general ward	Netherlands	To evaluate the effectiveness of continuous monitoring devices (ViSi Mobile and HealthPatch) in detecting clinical deterioration earlier than traditional nurse measurements, to compare the differences in MEWS results between device and nurse measurements, and to identify high MEWS periods that occur between regular nurse observations. The researchers wanted to find out if these devices could provide more accurate monitoring of vital signs and detect clinical deterioration at an earlier phase during unobserved periods	Randomised controlled trial, conducted prospectively	Surgical and internal medicine wards	Patients: 60 ViSi Mobile: 30, HealthPatch: 30 Control group: 30 (excluded from further analysis) The study focused on hospitalised non‐ICU patients	ViSi Mobile group: age median: 63 years (range 26–76) Gender male: 18 (60.0%) Female: 12 (40.0%) HealthPatch group: age median: 56 years (range 27–88) Gender: male: 22 (73.3%), female: 8 (26.7%)
65	Weenk et al.	2017	Continuous monitoring of vital signs using wearable devices on the general ward: Pilot study	Netherlands	To test the feasibility of continuous monitoring using the ViSi Mobile and HealthPatch devices and to evaluate the experiences of patients and nurses with these devices on the general ward	Pilot study, conducted prospectively	Internal medicine and surgical wards	Patients: 20 10 in internal medicine ward 10 in surgical ward Relatives: 7 Nurses: 4 Patients included those undergoing elective gastrointestinal operations and those with conditions such as sepsis, arthritis, and blood pressure control	Age mean: 49.9 years (SD 13.4), range 33–82 years 20 Male: 13 Female: 7
66	Weller et al.	2024	A retrospective observational study of continuous wireless vital sign monitoring via a medical grade wearable device on hospitalised floor patients	United States	To assess the agreement of BioButton‐recorded vital sign readings with manually charted bedside readings. Secondary objectives include evaluating the frequency and timing of notifications, and the impact on clinical management and length of stay	Observational study conducted retrospectively	Edical and surgical ward	Patients in medical‐surgical units, with a total of 11,977 patient encounters monitored (7872 at site 1 and 4105 at site 2) Nurses were involved in monitoring these patients using the BioDashboard system	No information on age or gender
67	Youssef Ali Amer et al.	2020	Vital signs prediction and early warning score calculation based on continuous monitoring of hospitalised patients using wearable technology	Belgium, Netherlands	To investigate continuous monitoring of hospitalised patients' vital signs using wearable technology for real‐time early warning scores (EWS) estimation and time‐series prediction. The objectives include collecting continuously monitored data using wearable sensors, developing an algorithm for early identification of clinical deterioration, optimising the conventional EWS system, and exploring prediction possibilities for vital signs and EWS for specific prediction horizons	Interventional, international, multicentre study, conducted prospectively	Cardiology ward, postsurgical ward, dialysis ward	Cardiology patients: 52 at ZOL, 21 at CHU‐ postsurgical patients: 10 at aZM (3 excluded)‐ dialysis patients: 7 at CHU (2 excluded)Total participants: 90 (before exclusions)	No information on age or gender

**TABLE 4:** Overview of study designs, participant groups, and clinical settings represented across the included studies.

Category	Type	Count of studies
Study design	Observational studies	19
	Cohort studies	11
	Randomised controlled trials	7
	Interventional studies	5
	Qualitative studies	5
	Pre‐ and post‐implementation trials	5
	Mixed‐methods studies	4
	Validation studies	4
	Before‐and‐after studies	2
	Feasibility studies	2
	Proof‐of‐concept studies	1
	Case series	1
	Quality improvement projects	1
Country of origin	Netherlands	25
	United Kingdom	13
	United States	6
	Germany	5
	Israel	3
	Denmark	2
	Canada	2
	Sierra Leone	2
	China	1
	France	1
	Greece	1
	South Korea	1
	Lebanon	1
	Switzerland	1
	Turkey	1
	Uganda	1
	Vietnam	1
	Belgium	1
	Japan	1
Year of publication	2015	1
	2016	2
	2017	1
	2018	3
	2019	6
	2020	9
	2021	9
	2022	17
	2023	9
	2024	4
	2025	6
Participants	Patients	62
	Nurses	17
	Doctors	5
	Physician assistant	1
	Relative	1
Clinical settings	Surgical wards	38
	Medical wards	18
	Intensive care/Coronary care units	7
	Post‐Anaesthetic/Recovery units	6
	Emergency departments	5
	Oncology/Immunology/Haematology Wards	5
	Infectious disease wards	4
	COVID‐19‐specific Units	2
	Operating departments	2
	Palliative care	2
	Dialysis units	1
	Neuroscience ward	1
Vital signs monitored by wearable technology	Heart rate (HR)	57
	Respiratory rate (RR)	48
	Temperature (T)	30
	Peripheral oxygen saturation (SpO_2_)	27
	Blood pressure (BP)	19

#### 3.1.1 Summary of Study Characteristics

There was considerable methodological diversity, with observational and cohort designs being most common. Randomised controlled trials were rare (*n* = 7), and nearly a third (*n* = 21) were pilots, indicating an exploratory field. Sample sizes ranged from small feasibility (< 50) to larger cohort studies (> 500), mostly at single sites.

Patients were the primary participants, with a few studies including healthcare professionals, highlighting a gap in understanding the experiences of the nursing and medical workforce with wearable device monitoring. The clinical settings were diverse, comprising mainly medical and surgical wards, as well as specialised populations such as oncology, haematology, and individuals in epidemic settings (COVID‐19, Ebola). Participant demographics generally ranged from middle‐aged to older adults; some groups, like those undergoing cardiac surgery, were predominantly male. Nonetheless, inconsistent reporting of age, gender, and other sociodemographic details hindered the assessment of representativeness and equity. Geographically, the evidence reflects both global interest and uneven research activity. Most studies were conducted in high‐income countries, with the Netherlands (*n* = 25), the United Kingdom (*n* = 13), and the United States (*n* = 6) leading. Lower‐resource settings were also represented, including Uganda and Ebola treatment centres, as well as two multinational collaborations (UK/France; Netherlands/Belgium). This distribution highlights broad international engagement but also raises questions about the transferability of findings across diverse healthcare contexts.

There was an upward trend in research on wearable technologies for inpatient monitoring, with a surge in the early 2020s likely attributable to increased clinical interest, technological progress, and the acceleration of digital health adoption during COVID‐19. Recent years show a decline.

The physiological monitoring scope varied. Some devices tracked multiple parameters, but many focused on one. HR and RR were most frequently monitored, SpO_2_ and BP were less often measured, likely due to the technical limitations of wearable devices.

#### 3.1.2 Wearable Devices

The review highlighted a focus on patch‐based systems, although studies included various device types and manufacturers. Table 5 summarises these devices and the frequency with which they were included in studies. While some technologies were extensively evaluated, many ‘other’ devices appeared in only a single study, indicating device diversity but limited depth of evaluation. This uneven distribution underscores the need for further research across a wider range of wearable technologies. The emphasis on certain devices was influenced by regional adoption, including the popularity of patch‐based systems in the Netherlands and the UK. This geographic context adds important nuance to device distribution, suggesting that national healthcare infrastructure, regulatory environments, and local collaborations with manufacturers may have shaped research trends and device preferences.

**TABLE 5:** Wearable monitoring devices identified.

Device name	Manufacturer	Device type	No of paper mentions
Sensium vitals	The surgical company (TSC)	Patch	15
HealthPatch	Holst Centre	Patch	5
ViSi mobile	Soltera	Wrist band	9
Healthdot	Philips	Patch	6
Masimo radius−7 or Masimo safety NET	Masimo	Arm band	4
Biobeat	Biobeat	Patch and wristwatch	4
Biosensor	Philips	Patch	3
Apple watch 7 Fenix Pro Scan watch	Apple Garmen Withings	Smart watches	2
Everion	Biofourmis	Armband	2
Early sense	Baxter Hillrom	Under mattress	2
Fitness trackers (Fitbit Charge)	Google LLC	Wrist band	2
Vital Patch	MediBioSense	Patch	2
Other			15
Not provided	—	—	3

### 3.2 Thematic Synthesis

This review synthesised evidence into four themes that illustrate how wearable vital‐sign monitoring is evaluated and experienced across clinical, nursing, patient, and system contexts. The themes reveal patterns across various studies. Most evidence concentrated on clinical safety, particularly early detection of deterioration, while patient experience, organisational readiness, and resource impacts received less attention. Findings differed across themes in depth and significance.

Table 6 offers an overview of the distribution of evidence across themes and the nature of reported effects, serving as an evidentiary map to contextualise the thematic synthesis. The following narrative is an interpretive integration examining how findings cluster, diverge, and interact across themes to highlight both the potential benefits and limitations of wearable continuous monitoring in adult hospital in‐patients within modern nursing practice and health systems. Where studies reported evaluative outcomes, the impact is indicated as positive (+ve), negative (−ve), or mixed (±). In technical domains such as device accuracy, AI capability, and organisational readiness, reporting often lacked sufficient detail to determine impact; therefore, these areas are described according to whether they were investigated. For areas characterised by descriptive, technical, or exploratory findings, a checkmark (√) indicates that the outcome was covered, while a cross (X) denotes it was not examined.

**TABLE 6:** Evidence map summarising reported impacts of wearable continuous monitoring across clinical outcomes, nursing workflow, patient experience, and resource or organisational implications.

	Theme		Theme 1: Enhancing clinical safety through continuous monitoring			Theme 2: Transforming nursing practice and workflow integration			Theme 3: Patient experience of wearable monitoring			Theme 4: Resource implications and system‐wide impact
	Subthemes		Early recognition of physiological deterioration	Device accuracy and reliability	AI enabled prediction and data accuracy improvement	Workflow efficiency and workload distribution	Alert burden, meaning and professional response	Training, technical competence and organisational readiness	Patient Reassurance and empowering patients through continuous observation	Physical comfort, usability and autonomy	Patient anxiety, privacy and trust	Cost and value outcomes in the health system
1	Beaman, H., et al.	2023	X	√	X	+ve	X	X	X	±	X	X
2	Becking‐Verhaar, F. L., et al.	2023	+ve	√	X	±	‐ve	√	X	±	X	X
3	Breteler, M. J. M., et al.	2020a	+ve	√	X	+ve	‐ve	X	X	X	X	X
4	Breteler, M.J.M., et al.	2020b	X	√	X	X	‐ve	X	X	X	X	X
5	Breteler, M.J.M., et al.	2018	X	√	X	X	‐ve	X	X	X	X	X
6	Choi, A., et al.	2022	+ve	√	√	X	+ve	X	X	+ve	X	‐ve
7	Downey, C., et al.	2019	X	√	√	X	‐ve	X	X	X	X	X
8	Downey, C., et al.	2018	+ve	X	X	X	‐ve	√	+ve	+ve	X	+ve
9	Downey, C.L., et al.	2020	±	X	X	X	‐ve	√	+ve	±	X	+ve
10	Eddahchouri, Y., et al.	2023	X	X	X	±	X	X	X	X	X	X
11	Ghiasi, S., et al.	2022	X	X	√	+ve	X	X	X	+ve	X	X
12	Haahr‐Raunkjaer, et al.	2022b	X	X	X	X	‐ve	X	X	±	X	X
13	Haveman, M.E., et al.	2021	X	√	√	X	‐ve	X	X	+ve	X	X
14	Helmer, P., et al.	2025	X	√	X	X	X	X	+ve	+ve	X	X
15	Helmer, P., et al.	2024	X	√	X	X	X	X	X	X	X	X
16	Helmer, P., et al.	2022	X	√	X	X	X	X	X	X	X	X
17	Hernandez‐Silveira, M., et al.	2015	X	√	X	X	+ve	X	X	X	X	X
18	Iqbal, F.M., et al.	2022	X	X	X	±	‐ve	X	+ve	+ve	X	X
19	Ishikawa, M. & Sakamoto, A.	2021	+ve	√	X	+ve	‐ve	√	X	X	X	+ve
20	Itelman, E., et al.	2022	+ve	X	√	X	‐ve	X	X	X	X	X
21	Jacobs, F., et al.	2021	X	√	X	X	‐ve	X	X	X	X	X
22	Jacobsen, M., et al.	2022	X	X	√	X	X	X	X	+ve	X	X
23	Jokinen, J.D.V., et al.	2022	+ve	√	√	+ve	X	√	X	X	X	X
24	Joshi, M., et al.	2025	+ve	√	√	X	+ve	X	X	+ve	X	X
25	Joshi, M., et al.	2022a	±	X	X	±	‐ve	√	X	+ve	X	X
26	Joshi, M., et al.	2022b	+ve	X	X	±	‐ve	√	X	+ve	X	X
27	Joshi, M., et al.	2021	X	X	X	+ve	X	X	+ve	+ve	X	X
28	Kachel, E., et al.	2021	X	√	X	X	+ve	X	X	+ve	X	X
29	Kooij, L., et al.	2022	+ve	X	X	±	‐ve	√	+ve	‐ve	‐ve	+ve
30	Kroll, R.R., et al.	2016	X	√	X	X	X	X	X	X	X	+ve
31	Leenen, J.P.L., et al.	2024	+ve	X	X	+ve	‐ve	X	+ve	+ve	‐ve	+ve
32	Leenen, J.P.L., et al.	2023	±	X	√	‐ve	‐ve	√	X	X	X	X
33	Leenen, J. P. L., et al.	2022a	+ve	√	X	X	‐ve	√	X	+ve	+ve	X
34	Leenen, J.P.L., et al.	2022b	X	X	X	±	‐ve	√	X	X	X	X
35	Leenen, J.P.L., et al.	2021	X	√	X	‐ve	‐ve	√	+ve	+ve	X	X
36	Li, T., et al.	2019	X	√	X	X	X	X	X	X	X	X
37	Liu, Y., et al.	2020	+ve	√	√	+ve	X	X	X	+ve	X	+ve
38	Lockhorst, E. W., et al.	2025	+ve	√	X	+ve	‐ve	√	+ve	+ve	X	±
39	Logaras, E., et al.	2022	+ve	X	√	+ve	+ve	X	X	X	X	X
40	McGrath, S.P., et al.	2019	+ve	√	X	+ve	±	√	X	X	X	+ve
41	Murali, S., J. et al.	2020	X	√	X	X	+ve	X	X	+ve	X	X
42	Nantume, A., et al.	2022	±	√	X	+ve	‐ve	X	X	X	X	X
43	Page, B., et al.	2025	+ve	√	√	X	X	√	X	‐ve	X	‐ve
44	Patel, V., et al.	2022	X	X	X	‐ve	±	X	+ve	±	X	X
45	Peelen, R. V., H. et al.	2025	+ve	X	√	X	‐ve	X	X	X	X	X
46	Pietrantonio, F., et al.	2019	+ve	X	X	+ve	+ve	X	X	X	X	+ve
47	Posthuma, L.M., et al.	2020	+ve	√	X	X	‐ve	√	X	+ve	X	X
48	Reed, M.J., et al.	2018	+ve	X	X	+ve	‐ve	X	+ve	+ve	X	X
49	Restrepo, M., et al.	2021	X	√	X	±	X	√	+ve	±	X	X
50	Sharabi, I., et al.	2023	X	X	X	+ve	+ve	X	X	+ve	X	+ve
51	Steinhubl, S.R., et al.	2016	+ve	√	√	X	+ve	√	X	+ve	X	+ve
52	Stellpflug, C., et al.	2021	+ve	X	X	+ve	+ve	√	+ve	+ve	+ve	+ve
53	Tenge, T., et al.	2025	+ve	√	X	X	‐ve	X	X	+ve	X	X
54	Totuk, A., et al.	2023	X	√	X	X	X	X	X	X	X	+ve
55	Turan, A., et al.	2019	+ve	√	X	X	X	X	X	X	X	X
56	van der Stam, J.A., et al.	2023a	±	X	X	+ve	+ve	X	X	+ve	X	+ve
57	van Der Stam, J. A., et al.	2023b	±	√	X	+ve	X	X	X	X	X	X
58	van Goor, H.M.R., et al.	2021	X	X	X	±	+ve	√	X	X	X	X
59	van Noort, H. H. J., et al.	2024	+ve	√	√	+ve	±	√	+ve	±	X	+ve
60	van Velthoven, M.H., et al.	2023	X	√	X	X	X	√	+ve	±	X	X
61	Vroman, H., et al.	2023	+ve	X	√	X	X	√	X	+ve	X	+ve
62	Watkinson, P.J., et al.	2020	+ve	X	√	+ve	+ve	√	X	‐ve	X	X
63	Weenk, M., et al.	2020	+ve	X	X	±	‐ve	√	+ve	‐ve	‐ve	+ve
64	Weenk, M., et al.	2019	+ve	√	√	X	X	X	X	‐ve	±	X
65	Weenk, M., et al.	2017	+ve	√	X	±	‐ve	√	+ve	+ve	X	X
66	Weller, G.B.	2024	+ve	X	X	+ve	+ve	X	X	X	X	+ve
67	Youssef Ali Amer, A., et al.	2020	+ve	√	√	+ve	X	√	X	+ve	X	X

In keeping with reflexive thematic analysis (Braun and Clarke 2022), the synthesis prioritises patterns of shared meaning across the body of evidence rather than exhaustively citing every included study within each analytic theme. Table 7 presents the thematic mapping, linking the main themes and subthemes with their associated initial codes and indicating how these were represented across the included studies, including the number of papers addressing each subtheme and the direction of reported impacts. The full scope of included evidence and study characteristics is detailed in Tables 6 and 7, ensuring transparency and completeness of reporting. This approach is consistent with the aims of scoping review methodology, which emphasises mapping and interpreting the literature rather than quantitative aggregation of findings.

**TABLE 7:** Mapping of themes, subthemes, and associated initial codes from the thematic synthesis, indicating the direction of reported impacts across included studies.

Main themes	Subthemes	Indicative/Initial codes	No. of studies addressing sub‐theme √ (*n*)	Positive impact + ve (*n*)	Negative impact ‐ve (*n*)	Mixed impact ± (*n*)
Theme 1: Enhancing clinical safety through continuous monitoring	Early recognition of physiological deterioration	Detecting deterioration between observation rounds; seeing trends rather than snapshots; identifying subtle physiological change; earlier‐than‐clinical recognition of risk; recognising deterioration overnight; making invisible deterioration visible; continuous vigilance replacing intermittent checks; temporal advantage of continuous data; alerting before clinician awareness; supporting escalation decisions; confirming clinical intuition with data; reducing reliance on manual observation timing; translating physiological change into action; linking early signals to earlier intervention; safety through anticipation rather than reaction	39	33	0	6
	Device accuracy & reliability	Trusting the numbers; questioning data credibility; motion artefact disrupting signals; losing data continuity; signal dropouts and gaps; device detachment or poor adhesion; calibration drift over time; differences between vital signs; reliability varying by patient characteristics; ambulation affecting signal quality; needing confirmation from manual checks; interpreting unstable readings; managing missing or noisy data; accuracy dependent on context; reliability as a workflow issue	39			
	AI‐driven prediction & data accuracy improvement	Predicting deterioration trajectories; combining multiple vital signs into risk; algorithmic early warning; filtering noisy data; removing low‐quality signal segments; reducing false alarms computationally; adjudicating alerts automatically; learning patient‐specific baselines; enhancing signal interpretability; managing data overload; supporting rather than replacing judgement; questioning algorithm transparency; trusting machine outputs; AI as sense‐making support; limits of model generalisability	19			
Theme 2: Transforming nursing practice and workflow integration	Workflow efficiency & workload distribution	Reducing routine bedside observations; shifting monitoring labour; redistributing nursing time; centralising surveillance work; task‐shifting between roles; gaining time for patient care; reducing night‐time disruptions; parallel workflows increasing burden; duplication of monitoring tasks; setup and maintenance work; managing devices as extra labour; documentation automation; technology creating new work; efficiency dependent on integration; workload felt differently across roles	36	22	3	11
	Alert burden, meaning and professional response	Being overwhelmed by alerts; ignoring non‐actionable alarms; losing trust in alerts; struggling to prioritise signals; alerts without clear action; cognitive burden of continuous data; alarm fatigue as erosion of judgement; differentiating real from spurious alerts; alerts disrupting workflow; managing uncertainty in alerts; needing context to interpret alarms; alert governance shaping response; alarm volume versus alarm meaning; surveillance becoming background noise; safety technology becoming risk	48	14	30	4
	Training, technical competence & organisational readiness	Learning to interpret continuous data; building confidence over time; relying on local experts; needing hands‐on support; trial‐and‐error adaptation; adjusting thresholds locally; training beyond device operation; normalising new ways of working; lack of preparation undermining trust; digital literacy shaping adoption; organisational support enabling use; governance enabling safe response; staffing to support continuous monitoring; readiness as collective competence; technology outpacing training	27			
Theme 3: Patient experience of wearable monitoring	Psychological reassurance and perceived empowerment	Feeling watched over; feeling safer knowing someone is monitoring; trusting staff to respond; reassurance through visibility; monitoring as comfort; confidence in early detection; feeling involved in care; understanding what the data mean; data prompting conversations; empowerment through explanation; reassurance without engagement; data without interpretation; trust dependent on response; monitoring as caring presence; safety as relational	17	17	0	0
	Embodied experience – comfort, usability, and autonomy	Comfort wearing the device; forgetting the device is there; skin irritation or discomfort; difficulty sleeping with sensors; freedom to move; reduced tethering to equipment; device interfering with movement; needing help with devices; managing charging and maintenance; physical autonomy versus control; mobility as independence; technology enabling normality; practical inconvenience shaping experience; acceptability tied to comfort; body–technology interaction	41	28	5	8
	Anxiety, trust, and privacy	Worry about abnormal readings; anxiety from seeing data; uncertainty about what numbers mean; trusting or doubting the system; concern when alerts seem wrong; reduced staff contact causing unease; feeling observed rather than cared for; trust built through explanation; reassurance undermined by false alerts; ambivalence about monitoring; privacy taken for granted; data ownership not discussed; surveillance versus care; emotional response to monitoring; trust as fragile	6	2	3	1
Theme 4: Economic and organisational consequences of wearable monitoring	System‐level and economic implications	Time savings as value; releasing nursing capacity; productivity rather than cost cutting; workforce sustainability; cost inferred not calculated; economic benefit assumed; implementation costs overlooked; maintenance costs invisible; training as hidden cost; value beyond financial metrics; sustainability as organisational resilience; technology supporting staffing shortages; return on investment unclear; efficiency framed as capacity; system value versus unit cost	21	18	2	1

#### 3.2.1 Theme 1: Enhancing Clinical Safety Through Continuous Monitoring

Theme 1 synthesises evidence on whether continuous wearable vital‐sign monitoring can enhance clinical safety in adult inpatient care. Across studies, safety benefits were primarily attributed to earlier recognition of deterioration, but were moderated by measurement accuracy, data continuity, and the ability to translate continuous data streams into actionable decisions (including through AI).

##### 3.2.1.1 Subtheme 1.1: Early Recognition of Physiological Deterioration

Early recognition was the most frequently examined subtheme (39 studies), with most reporting positive impacts (33/39). Compared with intermittent manual observations, wearables generally supported earlier detection of abnormal physiology and earlier escalation. Earlier recognition was reported across multiple clinical problems, including sepsis (reported 5–9 h earlier) (Choi et al. 2022; Posthuma et al. 2020), arrhythmias (Jokinen et al. 2022; Page et al. 2025; Weenk et al. 2017), fever (Liu et al. 2020), hypotension/respiratory depression (Ishikawa and Sakamoto 2021), postoperative complications (Lockhorst et al. 2025; Turan et al. 2019), and pain (Tenge et al. 2025). Alerts sometimes occurred well before clinical recognition (14–40 h) and often between routine observation rounds (up to 10 h before the next scheduled manual observations) (Itelman et al. 2022; Lockhorst et al. 2025; Weller et al. 2024).

Wearables also surfaced patterns that were often missed or incompletely captured in routine care, such as prolonged hypotension and respiratory instability (Posthuma et al. 2020; Turan et al. 2019; Weenk et al. 2019). Where monitoring was matched with adequate response capacity and escalation pathways, earlier recognition translated into earlier intervention (Peelen et al. 2025) and faster sepsis treatment (e.g., antibiotic administration: 626 min vs. 1012.8 min) (Downey, Randell, et al. 2018). Reported downstream benefits included fewer major complications (Pietrantonio et al. 2019), earlier and/or more frequent rapid‐response activation, fewer unplanned ICU transfers with shorter ICU LOS (Downey et al. 2020; Leenen et al. 2024; Vroman et al. 2023) and shorter overall hospital stays (Weller et al. 2024).

Qualitative findings aligned with these results: clinicians described improved real‐time insight and greater confidence in recognising deterioration, particularly valuing trend displays and automated alerts for sense‐making and prioritisation (Becking‐Verhaar et al. 2023; Joshi, Archer, et al. 2022; Kooij et al. 2022; Weenk et al. 2020). Mixed findings (six studies) were most often explained by implementation frictions, delayed responses, false alerts, and technical/device problems, rather than the absence of early‐warning capability (Downey et al. 2020; Joshi, Ashrafian, et al. 2022; Leenen et al. 2023; Nantume et al. 2022; van der Stam, Mestrom, Nienhuijs, et al. 2023; van der Stam, Mestrom, Scheerhoorn, et al. 2023). A small number of studies (e.g., van der Stam, Mestrom, Nienhuijs, et al. 2023; van der Stam, Mestrom, Scheerhoorn, et al. 2023) found performance comparable to existing early warning approaches, without clear gains in earlier recognition. Overall, the evidence suggests that wearables can advance recognition, but the clinical impact depends on response readiness, alert governance, and system reliability.

##### 3.2.1.2 Subtheme 1.2: Device Accuracy and Reliability

This subtheme (39 studies) addresses whether wearable measures are accurate and reliable enough for inpatient use and what constrains performance in real‐world wards. Most studies used agreement/validation approaches (commonly Bland–Altman bias and limits of agreement), correlations, and indicators of artefact burden and data completeness. Others described “real‐world” reliability through pragmatic issues (connectivity, adhesion, calibration, and dropouts) that shape usable monitoring time even when point accuracy is acceptable.

Across platforms, heart rate showed the most consistent acceptable agreement (Choi et al. 2022; Haveman et al. 2021; Helmer et al. 2022, 2024, 2025; Jacobs et al. 2021; Kachel et al. 2021; Totuk et al. 2023; Weenk et al. 2017). In arrhythmia‐focused evaluations, HR performance varied across devices and sensing methods (e.g., electrocardiogram vs. non‐electrocardiogram), including underestimation on some platforms (Breteler, KleinJan, Numan, et al. 2020). Respiratory rate was more variable, with wider disagreement particularly in ambulatory patients and in the presence of motion artefact (Breteler et al. 2018; Downey et al. 2019; Li et al. 2019). Similarly, SpO_2_ performance was variable: some studies reported clinically acceptable accuracy, while others found poorer agreement than HR and/or greater device‐to‐device variability (Haveman et al. 2021; Helmer et al. 2024, 2025; Murali et al. 2020; Nantume et al. 2022; Restrepo et al. 2021; Totuk et al. 2023; Van Velthoven et al. 2023). Temperature also showed inconsistent agreement, including underestimation, and practical challenges (Haveman et al. 2021; Joshi et al. 2025; Leenen et al. 2021; Liu et al. 2020; Nantume et al. 2022; Steinhubl et al. 2016; Van Velthoven et al. 2023). Evidence regarding BP accuracy was context‐dependent, identifying issues with calibration and potential post‐calibration declines in some devices or implementations (Kachel et al. 2021; Turan et al. 2019; Van Velthoven et al. 2023).

Two reliability constraints repeatedly determined whether ‘continuous’ monitoring was truly continuous. First, motion artefact and signal‐processing limitations led to unstable readings and spurious outliers, especially for RR (Breteler et al. 2018; Downey et al. 2019; Hernandez‐Silveira et al. 2015). Second, interruptions caused by disconnection or dislodgement (Downey et al. 2019; Leenen et al. 2021), poor skin contact or adhesion (Murali et al. 2020; Page et al. 2025), battery life (Van Noort et al. 2024), and connectivity or transmission failures (Breteler, KleinJan, Dohmen, et al. 2020) reduced usable monitoring time and data continuity. Device performance also varied according to patient and contextual factors, including age, arrhythmias, obesity (Helmer et al. 2022), skin colour, sweating (Restrepo et al. 2021), and gender (Liu et al. 2020), reinforcing that ‘accuracy’ is not a fixed property but a situated one.

##### 3.2.1.3 Subtheme 1.3: AI‐Driven Prediction and Data Accuracy Improvement

Nineteen studies described AI in relation to wearable monitoring. Across these studies, AI use had three overlapping aims: (1) prediction/early warning and risk stratification from multivital trajectories, (2) anomaly detection and alerting relative to baseline, and (3) signal quality improvement (noise filtering, artefact reduction and removal of low‐quality segments). Predictive approaches generated early warning scores and were applied to outcomes including deterioration and sepsis‐related endpoints (Ghiasi et al. 2022; Logaras et al. 2022; Page et al. 2025; Watkinson et al. 2020). Some studies also described AI‐supported calibration (Peelen et al. 2025).

AI was also positioned as a mechanism for making continuous monitoring safer and more usable by improving signal quality and reducing false alarms. Studies described preprocessing and signal evaluation to reduce unusable data and stabilise continuous streams (Downey et al. 2019; Haveman et al. 2021; Steinhubl et al. 2016; Youssef Ali Amer et al. 2020). In temperature monitoring, AI methods were used to correct interference and estimate core temperature from peripheral measures (Liu et al. 2020). Several studies explicitly framed AI as a means of reducing false alarms via adjudication logic, machine‐learning alarm processing, and discarding low‐quality segments, including for rhythm anomaly detection and alarm adjudication (Jokinen et al. 2022; Joshi et al. 2025; Page et al. 2025; Steinhubl et al. 2016).

Across the AI literature, limitations were common, including small and imbalanced datasets (Ghiasi et al. 2022), study design (Choi et al. 2022), and challenges in data collection (Logaras et al. 2022). Overall, AI appeared most promising when it improved signal‐quality management and reduced spurious alerts, but its clinical value depended more on implementation and governance than on model performance alone. Taken together, these findings indicate that while wearable monitoring can enhance early recognition of deterioration, its safety impact is contingent on how continuous data are governed, interpreted, and acted upon in practice, dependencies examined in Theme 2.

#### 3.2.2 Theme 2: Workflow, Alert Load, and Implementation Preparedness

Theme 2 synthesises evidence on how wearable continuous monitoring reshapes nursing work, not by automating care, but by redistributing workload, redefining alert practices, and reorganising responsibility for surveillance and response. The findings highlight that clinical impact is shaped less by the technology itself than by how monitoring and alerts are integrated, governed, and enacted in practice.

##### 3.2.2.1 Subtheme 2.1: Workflow Efficiency and Workload Distribution

Across 36 studies, the effects were mixed: 22 reported benefits, 11 reported mixed outcomes, and 3 reported predominantly negative effects. Many studies have reported improved workflow efficiency when wearable monitoring replaced routine manual observations, particularly in overnight monitoring or in clinically stable patients.

Rather than reducing workload outright, wearable monitoring primarily redistributed cognitive, temporal, and technical labour. These redistributions shaped not only clinical responsiveness but also how monitoring was experienced by both staff and patients. Whether this redistribution was experienced as beneficial or burdensome depended less on the technology itself than on how monitoring reduced bedside visits and automated data capture, allowing nurses to focus on higher‐acuity care, patient interaction, pain management, and communication (Beaman et al. 2023; Breteler, KleinJan, Dohmen, et al. 2020; Ghiasi et al. 2022; Ishikawa and Sakamoto 2021; Jokinen et al. 2022; Lockhorst et al. 2025; Nantume et al. 2022; Stellpflug et al. 2021; van der Stam, Mestrom, Nienhuijs, et al. 2023, van der Stam, Mestrom, Scheerhoorn, et al. 2023; Watkinson et al. 2020). During the COVID‐19 pandemic, wearable monitoring enabled rapid identification of deterioration while reducing Emergency Department traffic and unnecessary staff exposure (Logaras et al. 2022).

Workflow gains were most apparent in settings where systems supported automated documentation and integration with electronic medical records, thereby reducing manual transcription, duplication, and time spent locating monitoring equipment (Jokinen et al. 2022; Liu et al. 2020; McGrath et al. 2019; Sharabi et al. 2023). Several studies described workload redistribution rather than reduction, including centralised monitoring models and task‐shifting between bedside and surveillance roles to support prioritisation and sustainability (Joshi et al. 2021; McGrath et al. 2019; Nantume et al. 2022; Van Noort et al. 2024; Weller et al. 2024). In some contexts, improved escalation pathways were associated with reduced physician workload and faster decision‐making (Leenen et al. 2024; Pietrantonio et al. 2019; Van Noort et al. 2024; Weller et al. 2024).

Negative or mixed impacts arose when wearable monitoring was layered onto existing practices without replacing them. Parallel workflows, such as continued manual observations alongside wearable monitoring, generated additional verification tasks, operational burden, and frustration (Leenen et al. 2021, 2023; Patel et al. 2022). Implementation demands related to device setup, calibration, connectivity, battery management, and alarm handling frequently offset efficiency gains, particularly where integration, training, or change readiness were limited (Becking‐Verhaar et al. 2023; Eddahchouri et al. 2023; Iqbal et al. 2022; Joshi, Ashrafian, et al. 2022; Kooij et al. 2022; Weenk et al. 2017, 2020). Overall, wearable monitoring improved workflow when it replaced manual observations and documentation, supported automation, and operated within clear escalation protocols; where it duplicated work, benefits were attenuated or reversed.

##### 3.2.2.2 Subtheme 2.2: Alert Burden, Meaning and Professional Response

Across 48 studies examining alerting, effects were predominantly negative (30 studies), with fewer reporting positive (14) or mixed (4) impacts. Alerts were more often found to generate workload, interruption, and cognitive burden than to provide clear or actionable clinical guidance. A consistent finding was that high volumes of false or non‐actionable alerts overwhelm healthcare professionals, increasing interruptions and workload (Breteler et al. 2018; Iqbal et al. 2022; Joshi, Archer, et al. 2022). Several studies reported that alert burden was sufficiently disruptive to act as a barrier to implementation. This burden led to behavioural adaptation; most concerningly, ignoring alarms, including those that could indicate clinical deterioration. Empirical studies explicitly linked frequent, low‐specificity alerts to alarm fatigue and reduced responsiveness (Haahr‐Raunkjaer, Mølgaard, et al. 2022; Haahr‐Raunkjaer, Rasmussen, et al. 2022; Leenen et al. 2021). In some cases, alarms were intentionally disabled or avoided because previous experiences showed unsustainable fatigue among nursing staff (Leenen et al. 2023, Leenen, Rasing, et al. 2022). Alarm fatigue was also associated with a decline in clinical judgement, with studies reporting poorer decision‐making among clinicians exposed to noisy vital‐sign data and frequent false alarms (Breteler, KleinJan, Numan, et al. 2020; Jacobs et al. 2021; Joshi, Ashrafian, et al. 2022). Trust in alerts appeared to be conditional rather than inherent. Studies showed that trust declined when alerts were perceived as inaccurate, excessive, or disruptive to workflow (Iqbal et al. 2022; Becking‐Verhaar et al. 2023), but improved when systems demonstrably reduced false alarms through artefact filtering and data‐quality measures (Hernandez‐Silveira et al. 2015). When alerting logic was transparent and aligned with nursing workflows, clinicians reported greater confidence in prioritising alerts (Posthuma et al. 2020; Logaras et al. 2022).

A key cross‐cutting finding was the difficulty in distinguishing real deterioration from spurious alerts, driven by artefacts and threshold‐based systems (Jacobs et al. 2021). This uncertainty increased cognitive burden and disrupted workflows and, in some studies, led to disengagement from the technology (Downey et al. 2020).

Conversely, studies reporting positive impacts consistently described active alert governance. Empirical reductions in alert volume, sometimes exceeding 90%, were achieved through threshold optimisation, persistence rules, and averaging windows, with corresponding improvements in usability and reduced fatigue (Downey, Randell, et al. 2018). Across the literature, governance mechanisms were the strongest determinant of whether alerts functioned as meaningful clinical signals (Pietrantonio et al. 2019) or became clinically irrelevant and degraded into background noise (Leenen et al. 2021).

Overall, the evidence suggests that alerts do not fail because clinicians ignore them, but because poorly governed alerting systems produce excessive, low‐value signals, turning surveillance from a safety mechanism into a source of cognitive and organisational risk.

##### 3.2.2.3 Subtheme 2.3: Training, Technical Competence, and Organisational Readiness

A total of 27 studies addressed this theme. The ability to manage alert burden and workflow change depended heavily on training, technical competence, and organisational readiness. Although training was frequently acknowledged as essential, it was often poorly specified. Where described, training ranged from brief onboarding sessions to more sustained implementation support through superusers, IT teams, and research staff providing bedside coaching and troubleshooting (Downey, Chapman, et al. 2018, Downey, Randell, et al. 2018, Downey et al. 2020; Joshi, Ashrafian, et al. 2022; Kooij et al. 2022; Posthuma et al. 2020; Steinhubl et al. 2016; Stellpflug et al. 2021; Van Goor et al. 2021; Watkinson et al. 2020). Such supports enabled phased rollouts, iterative refinement of alerts and workflows, and growing confidence in interpreting continuous data (Becking‐Verhaar et al. 2023; Leenen, Rasing, et al. 2022; Leenen et al. 2023; Van Noort et al. 2024).

Competence developed incrementally and was closely linked to the management of operational burdens, such as false alarms, workflow disruptions, and device usability issues. Sites responded by adjusting thresholds, refining escalation processes, or relying on local expert support (Downey, Chapman, et al. 2018; Downey, Randell, et al. 2018; Joshi, Archer, et al. 2022; Restrepo et al. 2021). Usability challenges related to device design, connectors, reapplication, and maintenance reinforced the need for ongoing hands‐on support rather than one‐off training (McGrath et al. 2019; Stellpflug et al. 2021). Organisational readiness depended on reliable workflows, transparent governance, and sufficient staffing to respond safely to continuous data streams (Leenen, Rasing, et al. 2022; Van Noort et al. 2024; Youssef Ali Amer et al. 2020).

Overall, the impact of wearable monitoring on nursing work was contingent rather than inherent: benefits were realised where organisations actively supported workload redistribution and protected clinical judgement. At the same time, poor alert governance and inadequate preparation risked converting safety monitoring into cognitive and professional strain.

#### 3.2.3 Theme 3: Patient Experience of Wearable Monitoring

Patient experience was typically reported as a secondary outcome, most often focusing on comfort and usability (41), with fewer examining reassurance (17) or trust, anxiety, and privacy (6). As shown in Theme 2, wearable monitoring reshaped nursing workflows and alert practices; Theme 3 examines how these organisational changes were experienced by patients, particularly with respect to reassurance, comfort, trust, and autonomy. Patient accounts also revealed a persistent duality: wearable monitoring could be experienced as both a form of care and a mode of observation, with reassurance contingent on confidence that data were actively attended to and enacted as care rather than passive surveillance.

##### 3.2.3.1 Subtheme 3.1: Psychological Reassurance and Perceived Empowerment

Patients consistently reported feeling reassured and safe while wearing monitoring devices, mainly because continuous observation increased confidence that deterioration would be detected and addressed promptly (Downey, Chapman, et al. 2018, Downey, Randell, et al. 2018, Downey et al. 2020; Joshi et al. 2021; Leenen et al. 2021, 2024; Lockhorst et al. 2025; Van Velthoven et al. 2023). Reassurance was often relational rather than technological, reflecting trust that clinicians were monitoring the data and responding appropriately.

Some studies have also reported psychological empowerment, particularly when patients can view or discuss their vital‐sign data with staff. Access to data supported understanding of illness trajectories, progress, and recovery, and, in some cases, encouraged engagement in care conversations (Reed et al. 2018; Restrepo et al. 2021; Van Noort et al. 2024). However, reassurance did not automatically translate into empowerment. Several studies have noted that data access alone is insufficient without explanation or support for interpretation, and that unclear or unexplained readings can hinder engagement rather than enhance it (Iqbal et al. 2022; Kooij et al. 2022). No study reported predominantly adverse effects on reassurance or perceived safety.

##### 3.2.3.2 Subtheme 3.2: Embodied Experience—Comfort, Usability, and Autonomy

Most studies reported that wearable devices were comfortable, lightweight, unobtrusive, and generally well tolerated, with minimal interference with sleep, mobility, or daily routines. These characteristics contributed to high acceptability and willingness to continue use across a range of clinical settings and patient groups (Choi et al. 2022; Ghiasi et al. 2022; Downey, Randell, et al. 2018; Joshi et al. 2021; Joshi, Ashrafian, et al. 2022; Joshi, Archer, et al. 2022; Helmer et al. 2025; Steinhubl et al. 2016; Tenge et al. 2025). Wearables were frequently perceived as less restrictive than traditional bedside monitors, supporting physical mobility and reducing feelings of tethering.

Negative or mixed experiences generally stemmed from practical and usability issues rather than rejection of continuous wearable monitoring itself. Reported problems included skin irritation (Downey, Randell, et al. 2018; Downey et al. 2020), discomfort when lying on the sensor (Leenen et al. 2024), device bulk (Becking‐Verhaar et al. 2023), frequent reapplication due to poor adhesion (Murali et al. 2020; Page et al. 2025; Restrepo et al. 2021), and technical issues such as charging (Haveman et al. 2021; Jacobsen et al. 2022) or connectivity (Weenk et al. 2017). These problems sometimes disrupted sleep or required staff help, reducing perceived convenience. In some studies, the burden of device management and false alerts also indirectly affected patient experience by impacting staff responsiveness and communication.

Autonomy was primarily experienced as physical independence rather than decisional control. While wearables enabled freer movement and reduced attachment to bedside equipment (Helmer et al. 2025; Joshi et al. 2025), gains in perceived autonomy were not universal. Several studies reported that improved comfort and mobility did not necessarily enhance patients' sense of control or independence, particularly when they lacked understanding of the data or how it informed care decisions (Kooij et al. 2022; Van Noort et al. 2024).

##### 3.2.3.3 Subtheme 3.3: Anxiety, Trust, and Privacy Concerns

Across the limited number of studies addressing anxiety, patients generally reported low distress associated with wearable monitoring, particularly when monitoring was linked to visible clinical oversight and timely response (Leenen, Rasing, et al. 2022; Stellpflug et al. 2021). When anxiety was reported, it was typically situational and related to viewing abnormal readings, uncertainty about the meaning of data, or concerns about discharge rather than continuous monitoring itself (Van Noort et al. 2024; Weenk et al. 2020). Quantitative assessments showed no significant differences in anxiety scores between intervention and control groups in at least one study (Weenk et al. 2019).

Trust was more fragile when patients perceived monitoring data as unreliable or insufficiently acted upon. False alerts, limited feedback, or reduced interpersonal contact occasionally undermined confidence in both the technology and the care process (Kooij et al. 2022; Leenen et al. 2024). Privacy and data confidentiality concerns were not explicitly examined, reflecting a gap in the literature rather than evidence of their absence. Overall, patient experience depended less on the use of wearable monitoring than on how its observational capacity was translated into meaningful clinical care within therapeutic relationships.

#### 3.2.4 Theme 4: Economic and Organisational Consequences of Wearable Vital Signs Monitoring

Theme 4 examines the organisational and economic implications of wearable continuous vital‐sign monitoring, building on the clinical safety gains (Theme 1), workflow transformation (Theme 2), and patient experiences (Theme 3) described above. Rather than focusing on how work is performed, this theme considers what those changes enable or cost at system level. A single subtheme is presented because although 21 studies examined this theme, the current evidence base does not support finer economic distinctions: few studies conducted formal economic evaluations, instead relying on indirect indicators such as time savings, resource utilisation, or changes in service use.

Although 18 studies framed positive economic value, this was primarily through labour efficiency and capacity release rather than direct financial savings. Reduced time spent on routine observations and documentation was interpreted as enabling redeployment of nursing time toward higher‐value clinical activities rather than workforce reduction (Ishikawa and Sakamoto 2021; McGrath et al. 2019; Sharabi et al. 2023; Steinhubl et al. 2016; Weenk et al. 2020). For instance, Ishikawa and Sakamoto (2021) reported a 61.3% reduction in nursing workload when wearable continuous monitoring devices were used, while McGrath et al. (2019) reported about three nursing hours saved per day on a 36‐bed unit and Pietrantonio et al. (2019) reported that nurses saved 50–58 min per patient daily on monitoring activities.

Only two studies conducted explicit economic evaluations. Downey et al. (2020) reported a 69.9% probability that wearable monitoring was cost‐saving, with a 58% probability of improving quality of life, although the results were uncertain, and the mechanisms underlying cost savings were unclear. Vroman et al. (2023) reported approximately €622 savings per patient after accounting for system, maintenance, and biosensor costs; however, the retrospective design and absence of formal cost‐effectiveness or return‐on‐investment modelling limited the strength of conclusions.

Some studies suggested broader organisational or sustainability benefits, including device reusability, reduced infection transmission, and potential environmental advantages, although these outcomes were not quantified (Kroll et al. 2016; Liu et al. 2020; Sharabi et al. 2023). Evidence of adverse economic impact was rare but included reports of increased costs or unfavourable economic balance where implementation demands, device costs, or workflow disruption outweighed perceived benefits (Choi et al. 2022; Page et al. 2025).

In summary, Theme 4 suggests that the primary system‐level value of wearable monitoring lies in workforce efficiency, flexibility, and service resilience rather than consistent direct cost savings. In the context of global nursing shortages and rising care complexity, wearable monitoring may contribute to workforce sustainability and safer surveillance, even where economic benefits are indirect or not yet demonstrated. The reliance on proxy measures highlights the need for more rigorous, prospective economic evaluations alongside large‐scale implementation studies.

## 4 Discussion

This review synthesised evidence on wearable devices for continuous vital signs monitoring among adult hospital inpatients from 67 studies across 19 countries. Wearable monitoring consistently improved early detection of patient deterioration. It enhanced nurses' situational awareness, although effects on clinical outcomes like ICU and hospital LOS varied. Nurses valued these technologies for improving safety and efficiency only when they did not require concurrent manual monitoring of vital signs. Patients reported reassurance and comfort, despite occasional anxiety, discomfort, and privacy concerns. Economic benefits were promising but mainly qualitative, with costs rarely considered. Overall, wearable monitoring can significantly improve patient safety and workflow when supported by system integration, nurse training and leadership.

The mixed outcomes on LOS and ICU transfers are better understood through socio‐technical and systems perspectives. The Systems Engineering Initiative for Patient Safety (SEIPS) model (Carayon et al. 2006, 2020) shows that clinical effectiveness depends on device accuracy and technology's interaction with people, tasks, and organisational environments. Complex Adaptive Systems (CAS) theory (Glover et al. 2020) recognises that acute care settings are dynamic systems in which contextual and behavioural factors influence technology performance. Variations in outcomes reflect differences in workflow, escalation protocols, and staff confidence. Even accurate devices may fail if alarm fatigue, interoperability issues, or limited training hinder use. These perspectives situate wearable monitoring within a complex socio‐technical system with unpredictable human, organisational, and technical components that interact in unforeseen ways. Wearable monitoring can overcome limitations of manual vital sign measurement, which are susceptible to human error and bias (Granholm et al. 2016; Palmer et al. 2023). Reason's (1990) Swiss Cheese Model shows that errors often stem from systemic weaknesses such as high workloads and poor workflow design. Automating observations with wearables can reduce errors but introduces challenges in alert management, data reliability, and workflow. Effective implementation must optimise safety and usability, supported by proper infrastructure and governance.

From an implementation science perspective, the variable impact of wearable continuous monitoring observed across studies reflects differences not in technological capability, but in implementation, context, and system readiness. Using the Consolidated Framework for Implementation Research (CFIR), facilitators and barriers identified align with intervention characteristics (e.g., accuracy, alert burden), inner setting factors (e.g., workflow compatibility, staffing, digital infrastructure), and individual characteristics (e.g., digital literacy) (Damschroder et al. 2009). Consistent with CFIR, most studies prioritised clinical or process outcomes, underreporting critical implementation outcomes such as acceptability and sustainability, thereby limiting understanding of why benefits were realised in some contexts but not others. Implementation science also emphasises the importance of context and equity, highlighting that technologies introduced without attention to local resources, patient populations, and organisational capacity may exacerbate disparities rather than improve care (Brownson et al. 2021).

An emerging development in this evidence base is the integration of artificial intelligence (AI) into wearable monitoring. AI‐enhanced systems show potential to improve signal quality, predict deterioration, and reduce false alarms (Downey, Randell, et al. 2018; Watkinson et al. 2020; Weller et al. 2024). Such capabilities could ease nurses' cognitive load by filtering artefacts and prioritising meaningful alerts, aligning with global enthusiasm for AI in healthcare (World Health Organization 2021). Concerns remain, however, about transparency, accountability, and bias. For example, studies have reported reduced accuracy among individuals with darker skin pigmentation or in low‐resource settings, often due to sensor limitations (Nantume et al. 2022), raising equity issues. To ensure safe and equitable implementation, nurse leaders must guide adoption within strong governance frameworks, as emphasised by World Health Organization (2021) and NICE (Unsworth et al. 2021). Nurses as interpreters of digital data need training in digital and AI literacy to assess algorithmic outputs and maintain clinical judgement.

Wearable monitoring can transform nursing workflows by saving time, improving prioritisation, and increasing responsiveness through continuous data flow (Downey et al. 2020; McGrath et al. 2019). However, efficiency gains are often mitigated by dual‐system demands, device unreliability, and alert fatigue (Becking‐Verhaar et al. 2023). Nurses worry that automated monitoring may diminish relational care if it replaces bedside interactions. SEIPS explains these tensions, highlighting that workflow effectiveness depends on aligning people, technology, and organisational structures (Carayon et al. 2020). Normalisation Process Theory (May and Finch 2009) explains why adoption varies: ongoing use relies on staff understanding, engagement, and alignment with existing routines. Leadership fostering co‐design, reflection, and flexibility via transformational or distributed approaches (Bass and Riggio 2006; Spillane 2008) is key to integrating new technologies while respecting professional values. Nursing theories like Watson's (2008) Theory of Human Caring and Locsin et al. (2019) Technological Competency as Caring in Nursing view technology as an extension of, rather than a substitute for, caring practice.

Patient perspectives suggest that wearable monitoring can provide reassurance but may also generate discomfort or anxiety. While some patients value continuous monitoring for enhancing safety and supporting autonomy, others report heightened anxiety related to constant device awareness or the visibility of real‐time data (Becking‐Verhaar et al. 2023; McGrath et al. 2019). The Person‐Centred Practice Framework (McCormack and McCance 2016) proposes that technology contributes to positive care experiences when it supports meaningful engagement and partnership. In contrast, when wearables substitute rather than complement relational care, they risk undermining patients' sense of human connection. Kolcaba's (2003) Theory of Comfort and Mishel (1988, 1990) Uncertainty in Illness Theory suggest that patient comfort and psychological adaptation are shaped by effective communication and understanding. Consistent with these theories, this review found that uncontextualised physiological data can exacerbate anxiety, whereas clear explanations and patient education promote reassurance and empowerment. Together, these findings underscore the importance of supportive communication, intuitive interface design, and patient co‐design to ensure wearable technologies enhance both safety and well‐being.

The limited economic evidence suggests potential cost savings through shorter LOS and fewer ICU admissions, with some studies indicating significant financial benefits (Beard et al. 2023; Holmes et al. 2024). However, costs related to infrastructure, maintenance, and training were often underreported, and financial impacts inferred rather than directly measured. This mirrors previous reviews of digital health interventions, including the absence of standardised economic modelling and the challenge of applying traditional health economics frameworks to complex e‐health systems (Gentili et al. 2022; Huter et al. 2022). Policies such as the World Health Organization (2021) Global Strategy on Digital Health and NICE's Evidence Standards Framework (Unsworth et al. 2021), stress the importance of comprehensive cost‐effectiveness evaluations that include direct and indirect costs and broader impacts such as environmental sustainability and staff workload. Nurse leaders can lead these analyses, ensuring investments reflect long‐term value, equity, and workforce considerations.

These findings indicate that wearable monitoring success depends more on system integration, leadership, and the preservation of person‐centred values than technology alone. Effective adoption requires interoperability, robust training, alert management strategies, and leadership that promotes engagement and equity. As AI and digital technologies continue to evolve, nurses and nurse leaders must play a central role in shaping safe, ethical, and inclusive implementation, ensuring that digital innovation strengthens rather than fragments the human dimensions of care.

### 4.1 Strengths and Limitations

This review's strength lies in its broad scope, encompassing various devices, settings, and countries, enabling cross‐comparative analysis of challenges and outcomes. However, heterogeneity in study designs, device types, and outcomes, along with small pilot studies and brief follow‐ups, limited comparability. Economic evidence was limited, and experiential outcomes were reported inconsistently, affecting transferability.

Although formal quality appraisal was not undertaken, in line with Joanna Briggs Institute guidance for scoping reviews, it is important to acknowledge limitations in the robustness of the underlying evidence. The literature was dominated by observational studies, pilot and feasibility evaluations, and single‐site implementations, with relatively few randomised controlled trials. This reflects the emerging, implementation‐focused nature of wearable monitoring technologies but limits confidence in claims about downstream outcomes such as LOS, ICU transfers, or mortality. Reported benefits were often context‐dependent and shaped by workflow integration, response capacity, and organisational readiness rather than by technology alone. Consequently, findings on effectiveness and economic value should be interpreted cautiously, with conclusions understood to map patterns and evidence gaps rather than establish causal impact. The review included only articles published in English, which may cause language and publication bias. Excluding grey literature, to focus on validated evidence and manage study volume, may reduce detailed reporting of individual studies. Finally, rapid innovation in wearable technologies may affect the long‐term relevance of these findings.

### 4.2 Implications for Nursing Practice, Leadership and Scholarship

This review indicates that wearable continuous vital signs monitoring can support patient safety only when nurses are empowered to interpret and act on continuous data. Wearable monitoring must reshape, rather than replace, clinical judgement, requiring skills in trend interpretation, alert prioritisation, and timely escalation. Patient reassurance and trust were strongest when monitoring was enacted as responsive care, underscoring the importance of visible nursing engagement, clear communication, and protection of relational care. Wearable monitoring must therefore be integrated in nursing practice in ways that mitigate alert fatigue and data overload while preserving professional accountability and patient‐centred care.

The review findings highlight a vital role of nursing leadership in shaping whether wearable monitoring enhances or undermines care delivery. Leadership decisions regarding workflow integration, alert governance, staffing models, and training determine whether wearable technology redistributes work effectively or increases cognitive load. Considering the limited and proxy‐based economic evidence, leaders should prioritise evaluations that include workforce sustainability, skill‐mix flexibility, and long‐term system value rather than short‐term cost savings. Transformational leadership that encourages co‐design and equity can help ensure digital and AI‐enabled monitoring strengthens professional judgement and maintains the human dimensions of nursing practice. This review extends nursing scholarship by conceptualising wearable continuous monitoring as a transformation of core nursing practices rather than a purely technical intervention. Continuous data reconfigure nursing surveillance from intermittent observation to sustained, technology‐mediated vigilance, requiring nurses to exercise professional judgement in interpreting alerts, trends, and contextual cues rather than relying solely on automated outputs. These findings highlight the evolving nature of nursing expertise in digitally augmented care environments. Nurses, who are often at the frontline of interpreting monitoring outputs, must be equipped with training and clear governance frameworks to ensure that AI‐driven alerts are acted upon safely and equitably (Janes et al. 2025).

The review also identifies ethical and relational implications for nursing theory. Alarm fatigue, cognitive burden, and responsibility for continuous data may contribute to moral tension when organisational capacity does not align with monitoring demands. Patient experiences further reveal a tension between monitoring as reassurance and monitoring as surveillance, underscoring the importance of relational care, trust, and explanation. Future nursing research should therefore prioritise theory‐informed studies that examine how digital monitoring reshapes professional judgement, ethical responsibility, and nurse–patient relationships, ensuring that technological innovation supports the relational foundations of nursing practice.

## 5 Conclusion

This review indicates that wearable vital signs monitoring functions as a sociotechnical system whose impact depends on how it is embedded within nursing workflows, alert practices, and organisational structures. While continuous monitoring can support earlier recognition of deterioration, benefits are contingent on reliable data, timely human response, and effective alert governance. Rather than reducing work outright, wearable monitoring redistributes nursing labour, with implications for professional judgement, workload, and workflow sustainability. Patient reassurance and trust similarly depend on monitoring being enacted as responsive care rather than passive observation. At the system level, economic value is inferred mainly through proxy measures, suggesting that the primary benefit of wearable monitoring may lie in supporting nursing capacity and skill mix in contexts of workforce constraint. Future research should address patient‐centred outcomes, workforce effects, and robust economic evaluation to guide safe and sustainable implementation.

## Author Contributions

All authors have agreed on the final version and meet at least one of the following criteria (recommended by the ICMJE*): (1) substantial contributions to conception and design, acquisition of data, or analysis and interpretation of data; (2) drafting the article or revising it critically for important intellectual content.

## Funding

The authors have nothing to report.

## Disclosure

Permission to Reproduce Material: No previously copyrighted material requiring permission for reproduction has been used in this manuscript.

## Ethics Statement

This scoping review was conducted in accordance with the Joanna Briggs Institute methodology and reported using the PRISMA‐ScR checklist. The protocol was prospectively registered on the Open Science Framework (OSF; registration date: 18 June 2024; https://osf.io/3xfts/). As this study involved the review of existing literature and did not include human participants, ethical approval and patient consent were not required.

## Conflicts of Interest

The authors declare no conflicts of interest.

## Data Availability

All data included in this review are derived from previously published studies, which are fully referenced in the manuscript. No new datasets were generated or analysed.
